# Efficacy of Immersive Virtual Reality Combined With Multisensor Biofeedback on Chronic Pain in Fibromyalgia: A Pilot Randomized Controlled Trial

**DOI:** 10.1002/acr2.70048

**Published:** 2025-05-15

**Authors:** Luca Chittaro, Simone Longhino, Marta Serafini, Sofia Cacioppo, Luca Quartuccio

**Affiliations:** ^1^ Human‐Computer Interaction Lab, Department of Mathematics, Computer Science, and Physics University of Udine Udine Italy; ^2^ Rheumatology Division, Department of Medicine University of Udine Udine Italy; ^3^ Rheumatology Division, Department of Medicine University of Udine and Azienda Sanitaria Universitaria Friuli Centrale Udine Italy

## Abstract

**Objective:**

Fibromyalgia (FM) is a syndrome marked by chronic pain, fatigue, and mood disorders. Nonpharmacologic strategies are recommended to avoid overuse of opioids or nonsteroidal anti‐inflammatory drugs, but current approaches often provide limited relief. This study aimed to preliminarily assess the efficacy and feasibility of a new combined intervention of immersive virtual reality with multisensor biofeedback (IVR‐BF) in FM management.

**Methods:**

In this single‐center, pilot, open‐label, randomized controlled trial, adult patients with FM were randomly assigned 1:1 to either the treatment (TR) group, receiving IVR‐BF immediately, or a waitlist control (WL) group, receiving IVR‐BF after the TR group completed treatment. The primary outcome was reduction in visual analog scale (VAS) pain scores in the TR group, after five IVR‐BF sessions, compared to the WL group, after the waiting period. Secondary outcomes included improvements in FM impact (FM Impact Questionnaire [FIQ] score) and qualitative aspect of pain (Short‐form McGill Pain Questionnaire [SF‐MPQ] score). A longitudinal analysis was conducted across all patients to examine the trends in VAS pain, SF‐MPQ, and FIQ score during the trial.

**Results:**

Fifty patients were screened, and 20 female patients (10 TR and 10 WL) completed the trial and were analyzed. Those in the TR group showed significantly lower VAS pain scores compared to those in the WL group (*P* = 0.011), along with significant improvement in the FIQ score (*P* = 0.018). The longitudinal analysis revealed progressive improvements in VAS pain, SF‐MPQ, and FIQ score, supported by physiologic improvements (heart rate variability, respiratory rate, skin conductance). No significant safety concerns were reported. Patients expressed a high level of satisfaction with the IVR experience.

**Conclusion:**

IVR‐BF is a feasible treatment that shows potential in reducing pain and improving quality of life in patients with FM, supporting the need for larger trials to further evaluate its efficacy.

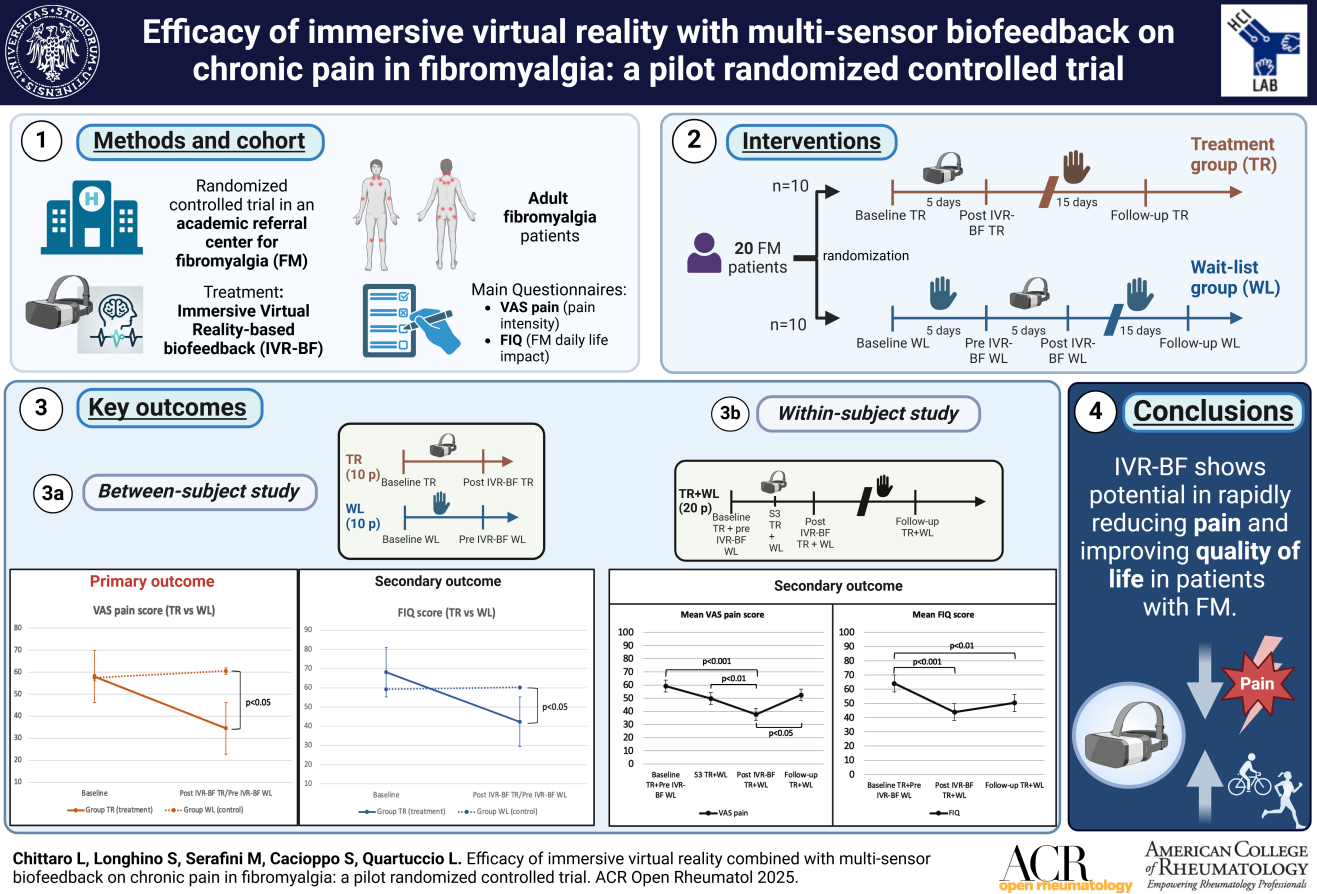

## Graphical abstract

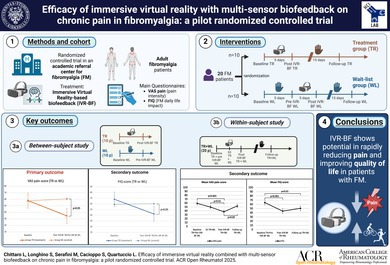

## INTRODUCTION

Fibromyalgia (FM) is a syndrome characterized by chronic musculoskeletal widespread pain, often accompanied by fatigue, sleep disturbances, cognitive impairment, and mood disorders.^1^ Its heterogeneous and debilitating symptomatology greatly reduces the quality of life for affected patients and leads to a substantial economic burden on society and health care systems, due to a high prevalence estimated between 2% and 8% worldwide.^2^, ^3^ Despite increased attention and progress in understanding FM, its management remains a pressing unmet need. Although a multimodal approach involving pharmacologic and nonpharmacologic strategies is encouraged in the treatment of patients with FM, it often provides only partial relief.^4^, ^5^, ^6^, ^7^, ^8^ In clinical practice, this situation often leads to widespread, erroneous, and dangerous prescription of analgesics, including opioids and nonsteroidal anti‐inflammatory drugs.^9^, ^10^

According to the latest Center for Disease Control and Prevention guidelines, nonpharmacologic approaches should be maximized before initiating opioid therapy for chronic pain.^11^ Therefore, there is a clear need for novel therapeutic approaches addressing the underlying mechanisms of FM and offering better symptom management. One such promising nonpharmacologic approach could involve biofeedback, which is a mechanism that enables individuals to become aware of and regulate their physiologic activity through the use of sensors, providing real‐time feedback and facilitating voluntary control of physiologic processes.^12^ Indeed, biofeedback exercises have shown potential in improving coping with stress—a central mechanism predisposing to nociplastic pain.^13^ The latest EULAR recommendations for FM management do not endorse biofeedback interventions due to limited evidence quality.^5^ Scarce user engagement due to abstract representations of physiologic signals can compromise biofeedback efficacy.^14^

A potential solution involves integrating biofeedback with visual immersive virtual reality (IVR), using a head‐mounted headset and headphones to immerse the patient in a virtual environment (VE).^14^, ^15^ Indeed, IVR has recently transitioned from a futuristic concept to a practical technology with significant therapeutic potential in pain management, offering immersive experiences that enhance cognitive distraction and reduce pain perception. If clinical trials consistently highlight IVR efficacy in alleviating acute pain, applying IVR for chronic pain, such as FM pain, is more complex due to the multifaceted nature of this condition.^16^ A preliminary study in rheumatologic setting showed promising results in pain reduction using an IVR system with respiratory biofeedback via a single sensor (microphone), but patients with FM were not included.^17^ A recent meta‐analysis of digital experiences in FM management has shown positive impact of virtual reality (VR), including reduced pain and fatigue, improved exercise capacity and quality of life, and better daily activity performance.^18^ However, most of the studies used nonimmersive VR, in which patients interact through a traditional desktop computer and monitor that do not isolate from the real world and thus immerse less in the experience. Moreover, none of them included biofeedback.

Therefore, the aim of our study is to assess, for the first time, the preliminary efficacy and feasibility of an IVR intervention combined with multisensor biofeedback (IVR‐BF) in reducing chronic pain intensity, modulating subjective sensory and emotional pain perception, and improving the overall impact of FM on daily functioning. In addition, we evaluated the efficacy of IVR‐BF in improving physiologic parameters associated with stress, that is, heart rate variability (HRV), respiratory rate (RR), and skin conductance.

## MATERIALS AND METHODS

### Study design

This pilot single‐center, open‐label, randomized controlled trial was conducted at a tertiary academic referral center for FM, with collaboration of the Human‐Computer Interaction Laboratory of the University of Udine. The study received approval from the institutional review board (131/2023) in accordance with the Declaration of Helsinki and followed the Consolidated Standards of Reporting Trials guidelines for randomized controlled trials.^19^ The trial was registered at ClinicalTrials.gov (identifier: NCT06100926) on 20 October 2023. The trial protocol is available in the Supplementary Materials.

### Participants

The study enrolled adult patients aged between 18 and 50 years with chronic nononcologic widespread musculoskeletal pain who met the 2016 Modified American College of Rheumatology (ACR) Diagnostic Criteria for FM.^20^ Exclusion criteria included concomitant diagnosis of (1) major psychiatric disorders, except for anxiety‐depressive disorder; (2) neurologic disorders, in particular those posing risk with IVR such as epilepsy; (3) autoimmune inflammatory rheumatic diseases causing chronic pain; (4) severe cardiac disease; (5) issues related to reality perception; and (6) substance addiction. These exclusions were made to avoid including patients unlikely to benefit or at risk from the intervention. All participants provided written consent.

### Randomization and masking

Participants were randomly assigned in a 1:1 ratio to either the treatment (TR) group or the waitlist control (WL) group. The TR group received the IVR‐BF intervention immediately, whereas the WL group received it after the TR group completed the treatment. Randomization was performed using automated software (StudyRandomizer.com) with a minimization algorithm to balance variables such as age, sex, marital status, comorbidities, employment, and ongoing treatments (Supplementary Table [Link], [Link]). The allocation was masked until the baseline assessment, with patients masked to group allocation. Investigators were aware of the group assignments as they administered the intervention.

### Study procedure

Each participant in the TR group completed five IVR‐BF sessions, with a maximum of one session per day, ideally finished within 10 days. During this time, patients in the WL group remained on a waitlist without treatment and served as controls. After the TR group completed all their sessions, the WL group received IVR‐BF following the same procedure. Both groups were monitored for up to 15 days after the final session. An overview of the study design is presented in Supplementary Figure S1. The choice to use a WL group is supported by the opportunity to apply two analytical approaches. The first is based on a randomized controlled trial design, comparing treated patients in the TR group with those in the WL group who waited without treatment in the first phase of the study (between‐subjects study, see Supplementary Figure S2). The second approach uses a before‐and‐after longitudinal design, considering all patients in both groups from the first session of IVR‐BF through the end of follow‐up to improve statistical power (within‐subjects study, see Supplementary Figure S3). This approach is also supported by previous publications on FM and IVR.^21^, ^22^, ^23^, ^24^

During the IVR‐BF session, the patient was comfortably seated in an armchair and equipped with a Meta Quest 2 headset (Meta). A Quest Touch controller (Meta) for use with the right hand was provided for a brief interaction with the VE that was required just once during the experience. To capture the physiologic data for the biofeedback, the patient wore a photoplethysmograph on the distal phalanx of the middle finger of the left hand to measure heart rate, two electrodes on the center of the palm and the carpus of the left hand to measure skin conductance, and an elastic girth sensor around the abdomen to assess respiratory activity. All physiologic sensors (Thought Technology) were recorded in real time using a Thought Technology ProComp Infiniti encoder at a sampling rate of 10 Hz. Each IVR‐BF session lasted 15 minutes and started with an initial phase that immersed the patient in a neutral VE representing a living room, whereas the baseline of physiologic activity was recorded for three minutes. Then, the patient performed diaphragmatic breathing for one minute, guided by a voiceover that provided instructions.

Next, the patient experienced a natural VE as illustrated in Supplementary Figure S4. The patient had to perform two tasks that change the appearance of the VE: (1) clearing the fog in the VE through slow, deep breathing (Task1), and (2) making the night fall by deeply relaxing, decreasing skin conductance (Task2). The two tasks lasted three minutes each. If the patient failed to complete them within this time frame, the system automatically completed them. During all the experience, the patient controlled different graphical elements of the environment through biofeedback. In particular, participants’ breathing affected the sound of wind blowing, the leaf swing of trees and bushes, the volume and pitch of the sound of windmill gears, and the rotation speed of windmill blades. During Task1, maintaining slow and deep breathing progressively pushed away the fog from the environment. During Task2, the participant made the night fall in the environment by relaxing: the position of the sun was controlled through skin conductance as a measure of participant's arousal. During this phase, fireflies in the environment glowed in synch with participant's heart rate. The two tasks were embedded in a story narrated by a voiceover, in which the participant is the main character who arrived in the Crystals Archipelago, a magical land that can connect with the user through magical crystals. A detailed description of the story and the implementation of the feedback mechanisms are provided in a technical article.^25^

During the study, questionnaires were administered at several time points, as illustrated by the detailed timeline in Supplementary Figure S1. The questionnaires were the (1) visual analog scale for pain (VAS pain), from 0 to 100, to assess pain intensity; (2) FM Impact Questionnaire (FIQ) to assess the impact of FM on daily life; (3) Short‐form McGill Pain Questionnaire (SF‐MPQ) to assess sensory and affective aspects of pain; and (4) Big Five Inventory (BFI) to assess personality traits. At the end of the study, satisfaction questionnaires about the IVR experience were administered to all patients.

The physiologic data collected during IVR‐BF sessions were skin conductance, RR, heart rate (HR), and HRV, expressed as root mean square of successive differences (RMSSD), number of pairs of successive heartbeat intervals differing by more than 50 milliseconds (NN50), and proportion of NN50 divided by the total number of heartbeat intervals (pNN50). These parameters were chosen because they are recognized as physiologic indicators of stress and pain.^26^ HRV was recorded at 500 Hz using a blood volume pulse finger clip sensor placed on the distal phalanx of the index finger of the left hand and connected to a BioSignalsPlux encoder (PLUX Biosignals). A sampling rate of 500 Hz is recommended for HRV analysis, as lower sampling rates can cause inaccuracy in HRV analysis.^27^

### Outcomes

The primary outcome of this study was to test the hypothesis of a significant reduction (at least 30%) in pain intensity, as assessed through the VAS pain, in the between‐subjects study among patients with FM in the TR group, after their five IVR‐BF sessions, compared to the patients with FM in the WL, after their waiting period (Supplementary Figure S2). Secondary outcomes included mitigation of FM impact on daily life, defined as a significant decrease in the FIQ score in the between‐subjects study among patients with FM in the TR group, compared to the patients with FM in the WL group, and improvement of sensory and emotional aspects of pain, assessed as reductions in sensory, affective, and total SF‐MPQ scores in the between‐subjects study among patients with FM in the TR group, compared to the patients with FM in the WL group.

A longitudinal analysis of VAS pain, SF‐MPQ, and FIQ scores was then conducted to examine temporal trends across all participants, starting from baseline for the TR group and before IVR‐BF for the WL group. This analysis extended to follow‐up assessment for both groups as presented in the within‐subjects study design (Supplementary Figure S3). Additionally, potential correlations between specific BFI personality traits and the response to IVR‐BF treatment were explored. Furthermore, because Task1 required patients to engage in a specific physiologic activity (slow, deep breathing) with the VE changing to reinforce that breathing activity, we performed an analysis of the physiologic biofeedback data collected by the IVR‐BF system during the performance of Task1 in each session.

Adverse events were systematically monitored throughout the study. Fifteen minutes after each IVR‐BF session, participants were asked to report any discomfort or side effects, including nausea, dizziness, fatigue, headache, visual disturbances, or any discomfort associated with the VR equipment. Investigators also observed participants during sessions for visible signs of distress or intolerance. Additionally, at the end of the follow‐up period, participants were asked about any persistent adverse effects attributed to the IVR‐BF treatment during this period. All reported adverse events were categorized by severity using a Likert scale (slight, moderate, and severe).

Finally, patient satisfaction with the IVR‐BF experience was assessed using a semistructured questionnaire at the end of the follow‐up period or on study discontinuation if participants chose to withdraw. The open‐ended responses were then analyzed to identify recurring concepts and organized into thematic categories by the investigators. This questionnaire was not mandatory and is available in Supplementary Materials.

### Statistical analysis

To detect a significant effect in the primary end point, a sample size of 20 patients (10 patients per group) was required. The sample size was determined using PASS software (v19.0.4), with an α of 0.05, power of 0.8, an expected VAS pain score of 67.11 (SD 14.88) at baseline, and an anticipated 30% reduction in the TR group compared to the WL group. Values were derived from studies on duloxetine, pregabalin, and milnacipran, US Food and Drug Administration (FDA)–approved for FM pain.^8^, ^28^ A per‐protocol analysis was conducted. The per‐protocol set was predefined to include only participants who strictly adhered to the assigned intervention without major protocol deviations. Specifically, patients were included in the study only if they completed all five IVR‐BF sessions within 10 days from the start and responded to all required questionnaires at the predefined time points, except for the satisfaction questionnaire, which was optional. Technical issues related to the recording of physiologic signals for the analysis of physiologic biofeedback data in Task1 did not lead to patient exclusion from the study. Patients were not allowed to initiate any concomitant pharmacologic or nonpharmacologic treatment for FM from enrollment until the end of the study, as this would result in exclusion. Major deviations, such as early discontinuation or noncompliance with the IVR protocol, led to exclusion from the study. This approach ensures a rigorous assessment of treatment effects while maintaining internal validity.

Given the small sample size, baseline characteristics of patients in the TR and WL groups were compared to assess statistically significant differences. Student's *t*‐test or Mann‐Whitney U‐test was used for continuous variables, depending on the normality of the distribution as determined by Shapiro‐Wilk test. For categorical variables, Fisher's Exact test was applied.

A 2 × 2 mixed design analysis of variance (ANOVA) compared VAS, SF‐MPQ, and FIQ scores between groups (TR and WL) and across time (before and after treatment). Bonferroni correction was applied for significant main effects. The percentage change from baseline to after treatment was compared using Student's *t*‐test. Next, we aggregated data from the two groups (TR and WL) to form a single group, comparing the questionnaires scores over time with a repeated‐measures ANOVA, starting from baseline for TR group and before IVR‐BF for WL group, until follow‐up assessment for both groups. Greenhouse‐Geisser estimates corrected for possible sphericity violations. Moreover, Pearson correlation assessed relationships between BFI traits and score changes between after treatment and before treatment of the VAS pain, SF‐MPQ, and FIQ. Physiologic data collected during baseline and Task1 were preprocessed using NeuroKit Python library to extract the tonic (skin conductance level [SCL]) component of skin conductance, and deriving the HRV measures (RMSSD, NN50, and pNN50).^29^ For each session, the Shapiro‐Wilk normality test was applied to baseline and Task1 data; nonnormal data were log or square‐root transformed.^30^ Extreme outliers were excluded from the analyses. A repeated‐measures ANOVA compared baseline and Task1 averages. All analyses were conducted using SPSS v29.0.0.0. The data sets used and analyzed during the current study are available from the corresponding author on reasonable request for noncommercial purposes.

## RESULTS

### Enrollment and baseline data

**Figure 1:**
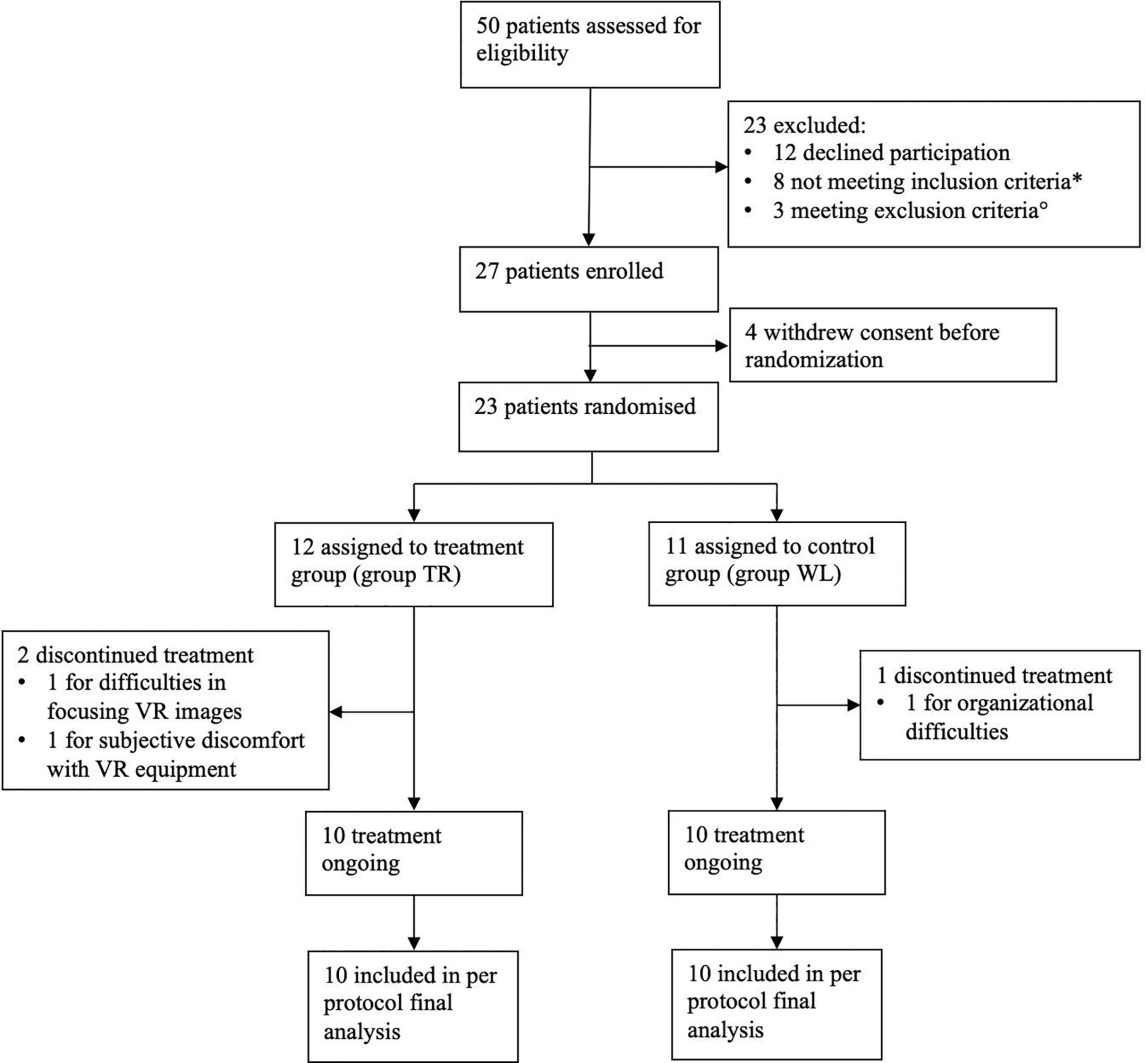
Consolidated Standards of Reporting Trials profile. Legend: *not meeting 2016 American College of Rheumatology Diagnostic Criteria for fibromyalgia; °the presence of a concomitant autoimmune inflammatory rheumatic disease was the exclusion criterion for all three patients. TR, treatment; WL, waitlist control.

From July 4, 2023, to September 9, 2023, 50 patients (48 women and 2 men) with a previous clinical diagnosis of FM were screened, and 27 were enrolled. Of the 23 of 50 patients who were excluded, 12 patients declined to participate, 6 patients did not meet the 2016 ACR diagnostic criteria for FM, including 2 men who also had a concurrent diagnosis of seronegative rheumatoid arthritis, and 3 patients were excluded due to a concomitant autoimmune inflammatory rheumatic disease. As a result, all 27 enrolled patients were women. After excluding four patients who withdrew consent before randomization, the remaining 23 patients were assigned to the two groups: 12 patients in the TR group and 11 patients in the WL group, with an overall median age of 47 (IQR 43–49) years and an overall mean VAS pain score of 58.91 (SD 19.77). Information about comorbidities, marital status, employment, ongoing pharmacologic and nonpharmacologic treatments, and baseline scores of VAS pain, FIQ, SF‐MPQ, along with BFI personality traits in the two groups are shown in Table 1. No statistically significant differences in baseline characteristics were noted between the TR and WL groups. During the trial, two patients dropped out from the TR group, one reporting difficulties in focusing images during the experience and one reporting discomfort with wearing the VR equipment; another patient dropped out from the WL group during the waiting period due to organizational difficulties. As shown in Figure 1, 20 patients were included in the final analysis (10 patients in the TR group and 10 patients in the WL group).

**Table 1:** Baseline characteristics of patients with fibromyalgia*

Characteristic	Treatment group (n = 12)	Control group (n = 11)	*P* value
Age, median (IQR), y	44.00 (41.75–47.75)	49.00 (47–49)	0.242
Sex, n (%)			>0.99
Female	12 (100)	11 (100)	
Male	0 (0)	0 (0)	
Comorbidity, n (%)			0.881
None	6 (50.00)	5 (45.45)	
Spondylarthrosis/peripheral arthrosis	3 (25.00)	2 (18.18)	
Depression	1 (8.33)	3 (27.27)	
Migraine	2 (16.67)	1 (9.09)	
Chronic pelvic pain	1 (8.33)	1 (9.09)	
Marital status, n (%)			0.265
Single	5 (41.67)	4 (36.36)	
Married	4 (33.33)	6 (54.54)	
Divorced	3 (25.00)	0 (0)	
Widow	0 (0)	1 (9.09)	
Employment, n (%)			0.232
Unemployed	0 (0)	3 (27.27)	
Physically demanding job	7 (58.33)	5 (45.45)	
Sedentary job	5 (41.67)	3 (27.27)	
Pharmacologic therapy, n (%)			>0.99
None	5 (41.67)	5 (45.45)	
Acetaminophen/NSAIDs	2 (16.67)	2 (18.18)	
Opioids/cannabinoids	1 (8.33)	1 (9.09)	
Antidepressants (duloxetine, amitriptyline)/antiepileptic	4 (33.33)	4 (36.36)	
Muscle relaxants	1 (8.33)	1 (9.09)	
Nonpharmacologic therapy, n (%)			0.680
None	6 (50.00)	4 (36.36)	
Mild physical activity	6 (50.00)	6 (54.54)	
Balneotherapy	0 (0)	1 (9.09)	
Ozone therapy	0 (0)	1 (9.09)	
Cognitive behavioral therapy	0 (0)	0 (0)	
Meditation	0 (0)	0 (0)	
Acupuncture	0 (0)	0 (0)	
VAS pain score, mean (SD)	58.75 (14.64)	59.09 (24.98)	0.484
FIQ score, mean (SD)	68.83 (11.82)	59.18 (15.61)	0.054
SF‐MPQ score, mean (SD)			
Sensory	15.58 (6.79)	12.82 (7.24)	0.178
Affective	7.33 (3.23)	5.73 (2.69)	0.106
Total	22.92 (9.61)	18.54 (9.32)	0.269
PPI	2.58 (1.08)	3.00 (1.00)	0.175
BFI score, mean (SD)			
Extraversion	27.50 (3.71)	26.18 (4.73)	0.231
Agreeableness	22.50 (3.80)	23.45 (4.34)	0.290
Conscientiousness	27.17 (5.20)	26.82 (4.45)	0.433
Neuroticism	28.08 (3.12)	26.82 (2.52)	0.150
Openness	37.33 (5.47)	34.09 (3.53)	0.055

* VAS pain, FIQ, Sensory SF‐MPQ, affective SF‐MPQ, total SF‐MPQ, PPI, extraversion, agreeableness, conscientiousness, neuroticism, and openness scores are normally distributed according to Shapiro‐Wilk test (*P* > 0.05); only age is not normally distributed according to Shapiro‐Wilk test (*P* < 0.001). Statistical test performed: Wilcoxon Mann‐Whitney for age; Fisher's exact test for sex, comorbidity, marital status, employment, and pharmacologic and nonpharmacologic therapy; Student's *t*‐test for VAS pain, FIQ, SF‐MPQ, and BFI scores. BFI, Big Five Inventory; FIQ, Fibromyalgia Impact Questionnaire; IQR, interquartile range; NSAID, nonsteroidal anti‐inflammatory drug; PPI, present pain index; SF‐MPQ, Short‐form McGill Pain Questionnaire; VAS, visual analog scale.

### Primary outcome

**Figure 2:**
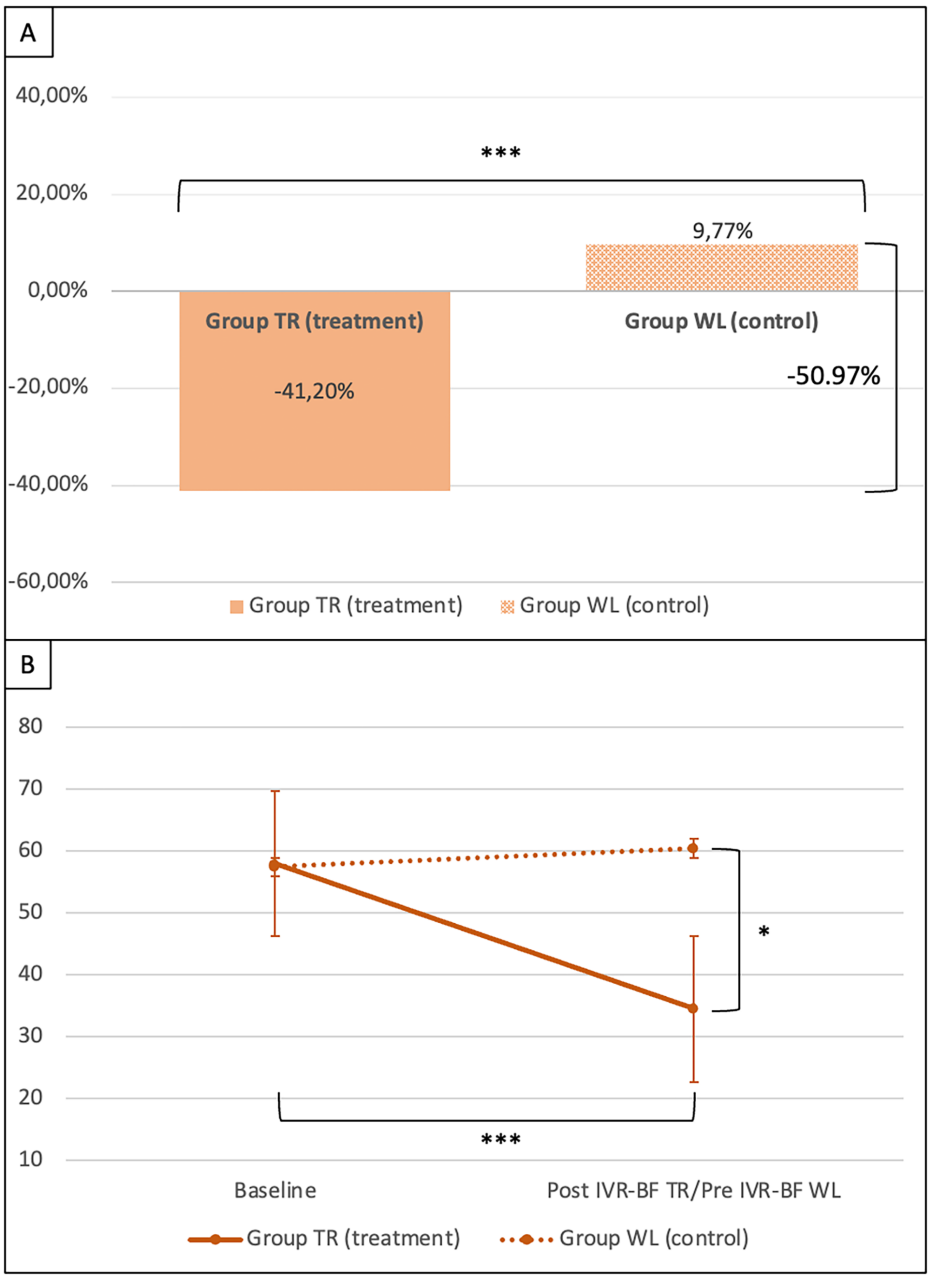
Visual analog scale (VAS) pain comparison between groups (TR group vs WL group). (A) Percentage of improvement of VAS score pain after IVR‐BF in the TR group compared to the WL group (***P < 0.001). Statistical test performed: Student's t‐test. (B) Improvement in VAS pain score in the TR group compared to the WL group (*P < 0.05) and improvement in VAS pain score in the TR group from baseline to the end of the IVR‐BF sessions (***P < 0.001). Statistical test performed: 2 × 2 mixed design analysis of variance (ANOVA) with Bonferroni correction. IVR‐BF, immersive virtual reality with multisensor biofeedback; TR, treatment; WL, waitlist control.

Considering the percentage change in VAS pain score at the end of the five IVR‐BF sessions for the TR group and at the end of the waiting period for the WL group, a significantly greater reduction in VAS pain was observed in patients who underwent IVR‐BF compared to those who were untreated (VAS t(18) = −4.04; *P* < 0.001). An overall VAS pain improvement of 50.97% was recorded in the TR group compared to the WL group (Figure 2A). Patients in the TR group also exhibited a significantly lower absolute VAS pain score after completing their five sessions of IVR‐BF compared to the patients in the WL group, at the end of their waiting period (mean ΔVAS −26.00, 95% confidence interval [CI] −45.17 to −6.83; *P* = 0.011). Moreover, patients in the TR group exhibited a significant improvement in VAS pain after the five sessions of IVR‐BF compared to their baseline (mean ΔVAS −23.50, 95% CI −32.24 to −14.77; *P* < 0.001); conversely, this improvement was not observed for the WL group (mean ΔVAS 3.00, 95% CI −5.74 to 11.74; *P* = 0.480) (Figure 2B).

### Secondary outcomes

**Figure 3:**
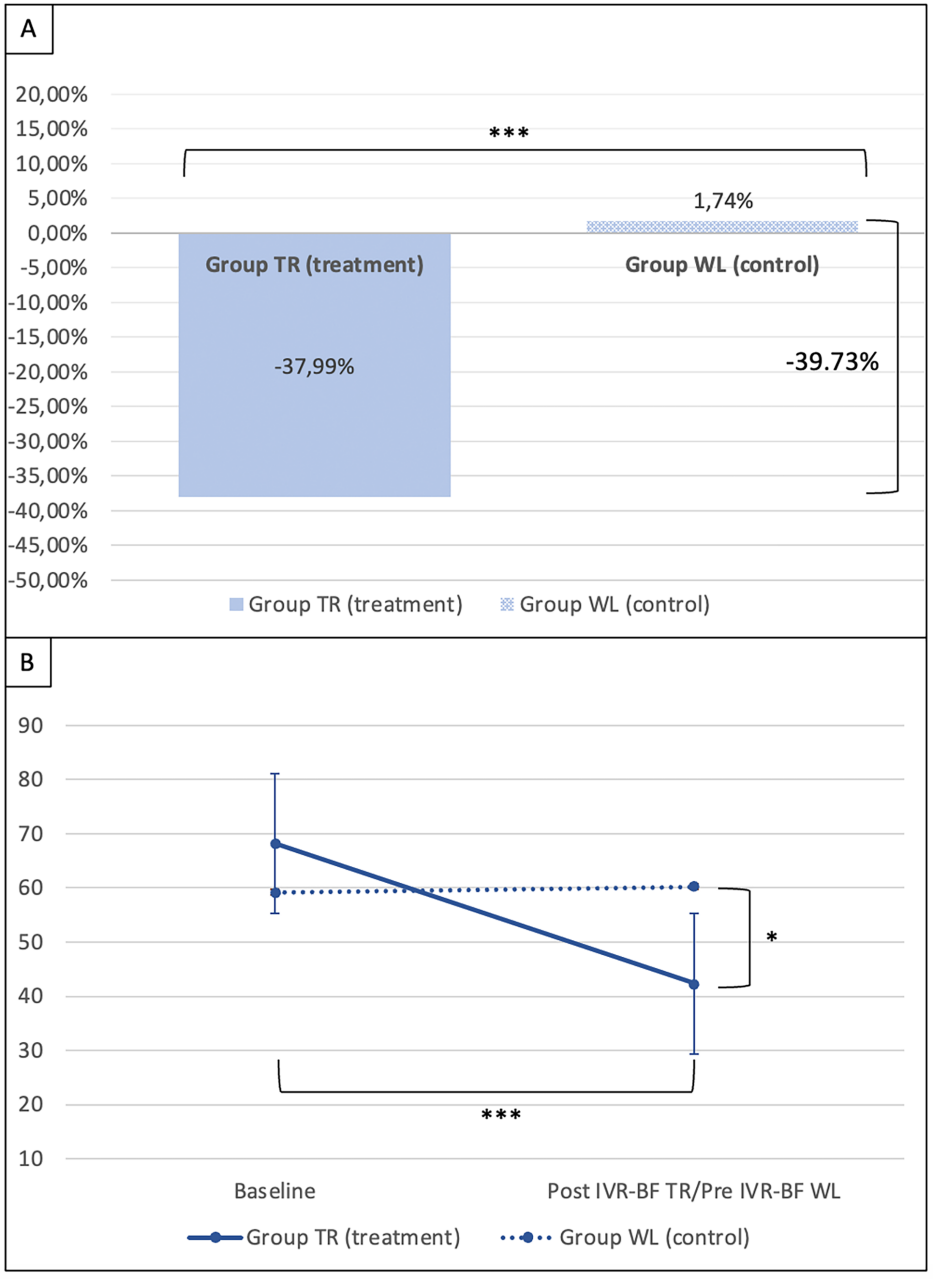
Fibromyalgia Impact Questionnaire (FIQ) score comparison between groups (TR group vs WL group). (A) Percentage of improvement of FIQ score after IVR‐BF in the TR group compared to the WL group (***P < 0.001). Statistical test performed: Student's *t*‐test. (B) Improvement in FIQ score in the TR group compared to the WL group (*P < 0.05) and improvement in FIQ score in the TR group from baseline to the end of the IVR‐BF sessions (***P < 0.001). Statistical test performed: 2 × 2 mixed design analysis of variance (ANOVA) with Bonferroni correction. IVR‐BF, immersive virtual reality with multisensor biofeedback; TR, treatment; WL, waitlist control.

Considering the percentage change in FIQ score at the end of the five IVR‐BF sessions for the TR group and at the end of the waiting period for the WL group, a significantly greater reduction in FIQ was observed in patients who underwent IVR‐BF compared to those who were untreated (FIQ t(18) = −6.20; *P* < 0.001). An overall FIQ score improvement of 39.73% was recorded in the TR group compared to the WL group (Figure 3A). This significant reduction in the impact of FM on daily life was further supported by the lower absolute FIQ score in the TR group at the end of the IVR‐BF sessions compared to the WL group at the end of the waiting period (mean ΔFIQ −17.90, 95% CI −32.37 to −3.43; *P* = 0.018). Additionally, patients in the TR group showed a significantly lower FIQ score after five sessions of IVR‐BF compared to their baseline (mean ΔFIQ −25.80, 95% CI −32.24 to −14.77; *P* < 0.001), whereas this improvement was not observed in the WL group (mean ΔFIQ 1.00, 95% CI −5.24 to 7.24; *P* = 0.740) (Figure 3B).

Regarding SF‐MPQ, no differences were found in sensory (mean ΔSF‐MPQ_sensory_ 3.80, 95% CI −1.65 to 9.25; *P* = 0.160), affective (mean ΔSF‐MPQ_affective_ 1.40, 95% CI −0.72 to 3.52; *P* = 0.182), total score (mean ΔSF‐MPQ_total_ 5.20, 95% CI −1.83 to 12.23; *P* = 0.14), and present pain index (PPI) (mean ΔSF‐MPQ_PPI_ 0.80, 95% CI −0.11 to 1.71; *P* = 0.082) following IVR‐BF sessions in the TR group compared to the WL group. A significant improvement was noted only in the affective domain for the TR group at the end of the five sessions compared to baseline (mean ΔSF‐MPQ_affective_ −2.70, 95% CI −4.45 to −0.95; *P* = 0.005) (Supplementary Figure S5).

**Figure 4:**
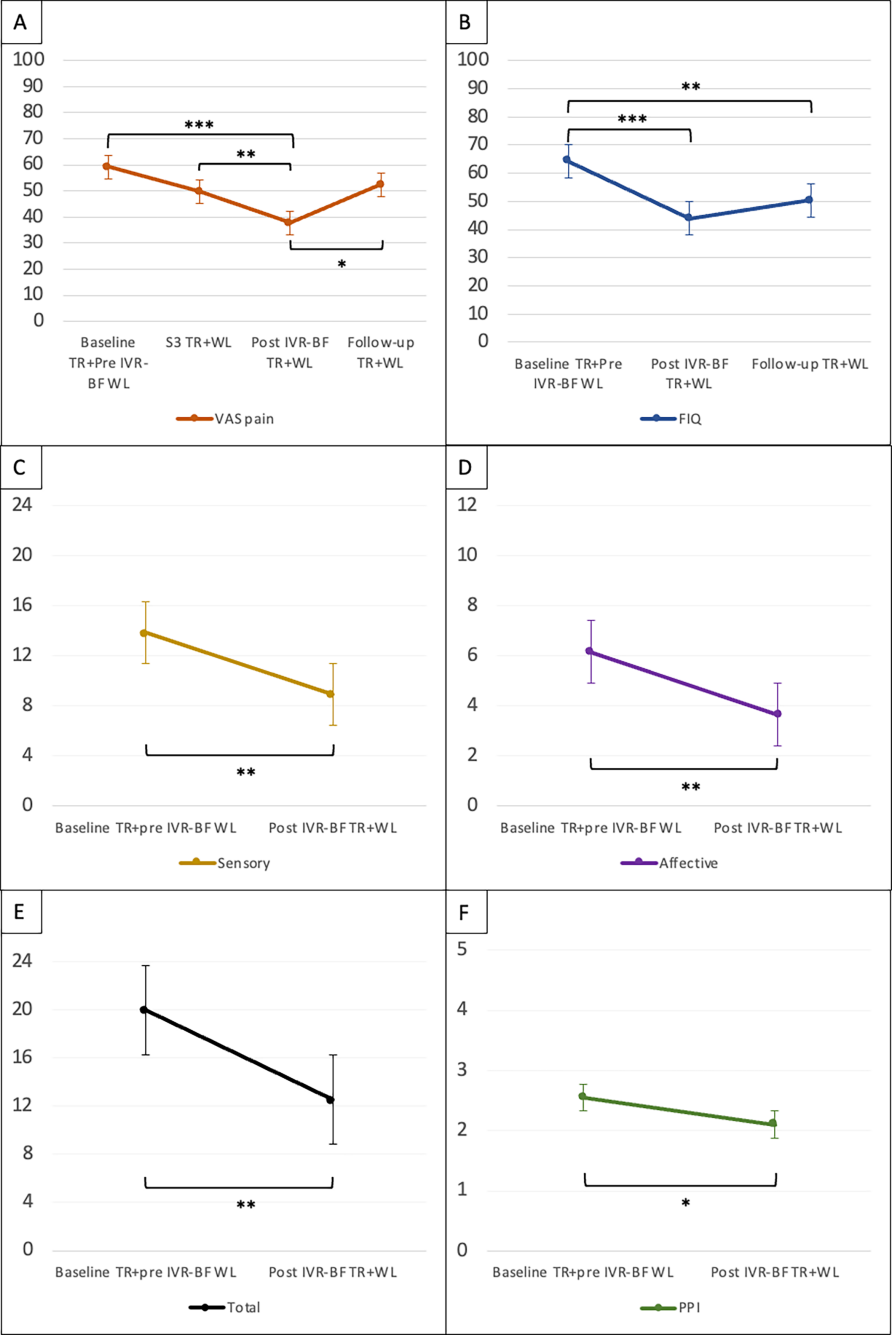
Mean VAS pain, FIQ, and Short‐form McGill Pain Questionnaire (SF‐MPQ) score trends during the study. Change in (A) VAS pain, (B) FIQ, (C) sensory SF‐MPQ, (D) affective SF‐MPQ, (E) total SF‐MPQ, and (F) PPI SF‐MPQ scores considering all the 20 patients who underwent IVR‐BF (TR group + WL group WL) (*P < 0.05, **P < 0.01, ***P < 0.001). Statistical test performed: repeated‐measures analysis of variance (ANOVA). FIQ, Fibromyalgia Impact Questionnaire; IVR‐BF, immersive virtual reality with multisensor biofeedback; PPI, present pain index; TR, treatment; VAS, visual analog scale; WL, waitlist control.

The longitudinal analysis of all 20 patients who completed the IVR‐BF intervention, from the start of their treatment to the end of the 15 days of follow‐up, revealed a trend of improvement in both pain intensity and the impact of FM on daily life across the five sessions. An initial improvement in VAS pain score was noticeable after three sessions (mean ΔVAS −9.50, 95% CI −19.79 to 0.79; *P* = 0.082), reaching significance by the end of the five sessions (mean ΔVAS −21.50, 95% CI −32.18 to −10.82; *P* < 0.001). However, at the end of the 15 days of follow‐up, VAS pain increased again, reaching preintervention levels (mean ΔVAS −6.75, 95% CI −17.12 to 3.62; *P* = 0.423) (Figure 4A). A similar trend occurred for FIQ score, with a significant reduction at the end of the five sessions (mean ΔFIQ −20.25, 95% CI −27.65 to −12.85; *P* < 0.001). Despite a slight increase, FIQ score remained significantly lower at the end of the 15 days of follow‐up compared to baseline (mean ΔFIQ −13.75, 95% CI −23.09 to −4.41; *P* = 0.003) (Figure 4B). Considering SF‐MPQ domains, significant improvements were found in sensory (mean ΔSF‐MPQ_sensory_ −4.95, 95% CI −7.77 to −2.13; *P* = 0.002), affective (mean ΔSF‐MPQ_affective_ −2.50, 95% CI −3.76 to −1.24; *P* = 0.001), total score (mean ΔSF‐MPQ_total_ −7.45, 95% CI −11.32 to −3.58; *P* = 0.001), and PPI (mean ΔSF‐MPQ_PPI_ −0.45, 95% CI −0.84 to −0.06; *P* = 0.025) (Figure 4C–F). Supplementary Table S2 summarizes results from between‐subjects and within‐subjects analyses. No correlations were found between the five BFI personality traits and VAS pain, FIQ, or SF‐MPQ scores (Supplementary Table S3).

Table 2 shows physiologic data analysis of Task1. Statistically significant reductions in mean RR (breaths per minute) were observed across all sessions, along with significant changes in mean SCL (microSiemens) from the third session onward. For HRV data, improvements were observed in mean RMSSD (in milliseconds) in the last two sessions, and in mean NN50 and pNN50 (percentage) in the second, fourth, and fifth sessions. No significant changes were found in mean HR.

**Table 2:** Repeated‐measures analysis of variance (ANOVA) results on physiologic data*

Measure	N	Mean Δ (Task1‐baseline)	95% CI	Main effect		
				F	*P* value	η_p_^2^
Session 1						
**RR, bpm**	**20**	**−7.36**	**−9.81 to −4.91**	**F(1, 19) = 46.32**	**<0.001**	**0.71**
SCL, μS	20	0.27	−0.88 to 1.42	F(1, 19) = 0.25	0.626	0.01
RMSSD, ms	16	4.85	−1.08 to 10.78	F(1, 15) = 3.04	0.102	0.17
NN50, n	16	6.69	−0.88 to 14.25	F(1, 15) = 0.085	0.372	0.05
pNN50, %	15	3.02	−0.32 to 6.37	F(1, 14) = 3.75	0.073	0.21
HR, BPM	18	1.05	−0.89 to 2.99	F(1, 17) = 0.26	0.620	0.02
Session 2						
**RR, bpm**	**19**	**−6.54**	**−8.35 to −4.74**	**F(1, 18) = 58.21**	**<0.001**	**0.76**
SCL, μS	20	−1.14	−2.39 to 0.11	F(1, 19) = 3.63	0.072	0.16
RMSSD, ms	15	6.29	−1.50 to 14.08	F(1, 14) = 3.00	0.110	0.18
**NN50, n**	**15**	**13.40**	**4.20 to 22.60**	**F(1, 14) = 13.87**	**0.002**	**0.50**
**pNN50, %**	**15**	**5.99**	**1.91 to 10.07**	**F(1, 14) = 13.74**	**0.002**	**0.50**
HR, BPM	18	1.30	−1.41 to 4.00	F(1, 17) = 1.01	0.330	0.06
Session 3						
**RR, bpm**	**19**	**−6.46**	**−8.28 to −4.64**	**F(1, 18) = 55.75**	**<0.001**	**0.76**
**SCL, μS**	**20**	**−1.12**	**−2.21 to −0.04**	**F(1, 19) = 4.74**	**0.042**	**0.20**
RMSSD, ms	15	4.91	−1.61 to 11.42	F(1, 14) = 0.94	0.348	0.06
NN50, n	14	8.79	−3.11 to 20.68	F(1, 13) = 0.40	0.539	0.03
pNN50, %	14	3.64	−1.54 to 8.81	F(1, 13) = 0.78	0.394	0.06
HR, BPM	18	0.45	−1.27 to 2.16	F(1, 17) = 0.60	0.448	0.03
Session 4						
**RR, bpm**	**17**	**−7.83**	**−9.39 to −6.28**	**F(1, 16) = 93.35**	**<0.001**	**0.85**
**SCL, μS**	**19**	**−1.21**	**−2.20 to −0.23**	**F(1, 18) = 6.71**	**0.018**	**0.27**
**RMSSD, ms**	**17**	**8.04**	**1.72 to 14.36**	**F(1, 16) = 8.27**	**0.011**	**0.34**
**NN50, n**	**16**	**10.19**	**1.69 to 18.68**	**F(1, 15) = 5.58**	**0.032**	**0.27**
**pNN50, %**	**16**	**5.04**	**0.82 to 9.26**	**F(1, 15) = 6.14**	**0.026**	**0.29**
HR, BPM	18	−0.37	−2.22 to 1.49	F(1, 17) = 1.31	0.268	0.07
Session 5						
**RR, bpm**	**19**	**−6.18**	**−8.53 to −3.82**	**F(1, 18) = 30.40**	**<0.001**	**0.63**
**SCL, μS**	**19**	**−1.53**	**−3.03 to −0.03**	**F(1, 18) = 4.60**	**0.046**	**0.20**
**RMSSD, ms**	**14**	**8.89**	**0.70 to 17.08**	**F(1, 13) = 5.26**	**0.039**	**0.29**
**NN50, n**	**14**	**9.14**	**2.28 to 16.00**	**F(1, 13) = 9.08**	**0.010**	**0.41**
**pNN50, %**	**13**	**3.07**	**0.64 to 5.50**	**F(1, 12) = 7.72**	**0.017**	**0.39**
HR, BPM	18	0.25	−1.07 to 1.57	F(1, 17) = 0.17	0.683	0.01

* Statistically significant results are reported in bold. BPM, beats per minute; CI, confidence interval; HR, heart rate; N, number of patients considered in the analysis excluding outliers and technical problems in signal recording; NN50, number of pairs of successive heartbeat intervals differing by more than 50 milliseconds; pNN50, proportion of NN50 divided by the total number of heartbeat intervals; RMSSD, root mean square of successive differences; RR, respiratory rate; SCL, skin conductance levels; Task1, clearing the fog in the virtual environment through slow, deep breathing.

### Safety and patient satisfaction with IVR‐BF treatment

As shown in Supplementary Figure S6, 18 of the 23 patients (78.25%) experienced no adverse events during IVR‐BF treatment. Two patients (8.7%) reported slight fatigue after each session, but this did not lead to treatment discontinuation. Two patients (8.7%) experienced discomfort with the equipment: one reported mild discomfort due to perception of IVR headset weight, whereas the other experienced a strong feeling of constriction, resulting in study withdrawal. Finally, one patient (4.35%) reported moderate blurred vision during and immediately after IVR‐BF sessions, leading to withdrawal after session 3. No persistent disturbances related to the IVR‐BF treatment were reported during follow‐up.

Twenty‐one of 23 patients completed the satisfaction questionnaire about the IVR‐BF experience; 15 patients (71.43%) reported being satisfied, 3 patients (14.28%) rated the intervention as neutral, and 3 patients (14.28%) expressed dissatisfaction. Regarding the IVR experience, six patients appreciated the sense of relaxation, five patients valued its novelty, and five patients appreciated its high engagement. Conversely, the IVR experience was perceived as too repetitive by five patients, too demanding for the time required by two patients, and too simple by one patient. Overall, 17 of 21 patients (80.95%) stated that they would recommend the experience to other people with FM. A more detailed report of the satisfaction questionnaire results is provided in Supplementary Figure S7.

## DISCUSSION

This study is the first to combine an IVR experience with multisensor biofeedback to patients with FM, showing feasibility and significant improvement in chronic pain and daily life of FM after only five treatment sessions. Regarding feasibility, our pilot trial was characterized by an efficient recruitment process and high intervention adherence. Within just two months (July to September 2023), we screened 50 potential participants, enrolled 27 patients (54% recruitment rate), and completed the trial with 20 patients (40% trial completion rate). These data were consistent with the recruitment and completion rates reported in previous trials assessing the efficacy and acceptability of nonpharmacologic treatments for FM, including attention‐based VR interventions and transcranial direct current stimulation.^31^, ^32^ Strong intervention adherence was supported by its good tolerability, with a relatively low dropout rate (13% vs approximately 20% in trials with duloxetine and milnacipran).^8^ The brief duration of IVR‐BF sessions (15 minutes per day for five days) likely minimized the patients’ time burden, whereas the randomization technique balanced the groups despite the small sample size, further validating the study design. Finally, the patient‐reported outcome measures used to assess various aspects of pain and quality of life in patients with FM were simple and quick to complete, although they were subject to high subjective and temporal variability due to external factors such as stress and anxiety, which may not necessarily be directly related to FM. Additionally, physiologic data recorded noninvasively during IVR‐BF sessions showed high interpatient variability, leading to the exclusion of outliers as shown in Table 2. Future larger trials should mitigate these issues with more frequent, digitally collected measurements, obtained not only during clinical visits but also at home, to better capture fluctuations over time.

Regarding the preliminary efficacy, the magnitude of pain relief achieved by IVR‐BF intervention is comparable to that of licensed drugs for FM, such as duloxetine, milnacipran, and pregabalin.^8^, ^28^ The results about the impact of FM on daily life, as assessed by the FIQ questionnaire, are consistent with those observed for VAS pain, indicating that IVR‐BF positively influences multiple clinical aspects of FM beyond just musculoskeletal pain.

Current EULAR guidelines suggest that biofeedback alone is not recommended for FM due to weak evidence and limited effectiveness restricted solely on pain intensity control. Moreover, previous biofeedback studies in FM have primarily concentrated on electromyographic and electroneurographic assessments, ignoring other physiologic signals such as RR and HR.^5^ Implementing biofeedback with an immersive, multisensor IVR system, as seen in this study, offers a new approach. This system allows patients to engage in a relaxing VE and practice exercises aimed at regulating physiologic processes linked to pain and stress, echoing the suggestions about the potential of IVR‐BF to modulate pain through cognitive distraction and immersive experiences.^16^, ^33^ Our findings are consistent with previous works, highlighting the potential role of IVR in enhancing traditional nonpharmacologic therapies, aiming to offer better control of FM‐related symptoms.^17^, ^34^, ^35^ Notably, the therapeutic benefits of IVR‐BF were found to be independent of the BFI personality traits, suggesting efficacy regardless of the patient's personality profile.

The longitudinal analysis of all 20 patients revealed rapid improvements in both pain intensity and the overall impact of FM during and immediately after IVR‐BF sessions. Although improvements in pain intensity diminished during follow‐up, benefits related to daily life impact persisted for up to two weeks after treatment. This pattern of symptom relapse is consistent with the chronic nature of FM, which often sees patients experiencing a recurrence of symptoms after ceasing treatments.^36^, ^37^ The sustained improvement in FIQ scores underlines the potential of IVR‐BF to provide comprehensive benefits, extending beyond pain relief to address the full spectrum of FM clinical manifestations. In this context, we deliberately chose a short follow‐up, given the pilot nature of our study and the innovative application of IVR‐BF in FM. This approach allowed us to assess the short‐term effects of the treatment on pain and quality of life while also providing preliminary insights into a potential retreatment timeframe, which our findings suggest being approximately two weeks. Because FM is a chronic condition requiring long‐term management, future IVR‐BF applications should explore longer follow‐ups (eg, three months) and maintenance sessions to assess whether these benefits persist over time.

In evaluating qualitative pain aspects via the SF‐MPQ, the TR group exhibited improvements in all domains, although only the affective domain change was statistically significant. Longitudinally, analyzing all patients revealed net improvements across all SF‐MPQ items after IVR‐BF treatment compared to baseline. This apparent contradiction suggests that although IVR‐BF effectively addresses qualitative pain aspects, the effects on pain intensity may be more immediate than those on emotional or affective components, which may not be as pronounced in just five sessions of IVR‐BF.

The study is pioneering in using multiple physiologic data alongside patient‐reported outcomes to assess a novel treatment for FM. Patients practicing slow and deep breathing exercises during IVR‐BF showed progressive physiologic improvements over time. Significant reductions in RR, HRV indicators, and SCL denote enhanced parasympathetic activity and better stress management.^38^ Given that poor stress management and sympathetic hyperactivation are often associated with chronic pain, these findings suggest that targeting these mechanisms could benefit FM treatment.^39^

Regarding safety, this study is the first to evaluate this aspect in the context of IVR‐BF applied to FM, reporting a high patient satisfaction and only a few transient adverse events (fatigue, discomfort with equipment, and blurred vision) that led to early treatment discontinuation in only two patients. This stands in contrast to the several adverse effects noted in trials of licensed pharmacologic treatments such as pregabalin, milnacipran, and duloxetine.^8^, ^28^ Furthermore, patients appreciated the novelty and relaxation provided by the IVR‐BF treatment, although some found it repetitive, suggesting future iterations could be customized to enhance engagement.

This study has limitations. Firstly, it is a pilot randomized controlled trial without a placebo control group, and there is potential investigator bias due to unmasked results. Although the concordance between clinical efficacy and physiologic data emerging from our study supports the findings, a randomized controlled trial comparing an IVR‐BF system to an IVR‐placebo (IVR with sham biofeedback) would help determine whether the observed improvements result from the combined effect of IVR‐BF or simply from immersion in a relaxing VE. A recent article by our group provided interesting insights in this regard.^25^ Using the same IVR system as in this study, it compared real (IVR‐BF) and sham (IVR‐placebo) biofeedback to evaluate its specific role in reducing anxiety in healthy volunteers. The results showed that although IVR with sham biofeedback (IVR‐placebo) helps lower anxiety, IVR with real biofeedback (IVR‐BF) enhances user engagement and leads to greater relaxation and less anxiety levels compared to IVR‐placebo. Based on these findings, it is reasonable to hypothesize that IVR‐BF offers greater benefits in the context of FM, as well. To confirm this, a randomized controlled trial should directly compare patients receiving IVR‐BF with patients treated with IVR‐placebo, using specific outcomes such as pain intensity reduction and quality‐of‐life improvement. Furthermore, to minimize bias, evaluators of the clinical outcomes should be masked to group allocation and treatment administration. This can be achieved by ensuring that those assessing clinical outcomes are not directly involved in delivering the intervention. Such measures would enhance the validity of the results and provide robust evidence into the role of IVR‐BF in the management of FM.

Other limitations of this study include its short duration, the small and predominantly female sample from a tertiary referral center, and the per‐protocol analysis, which may limit the generalizability of the findings. This approach is justified by the need for a preliminary assessment of the efficacy of IVR‐BF among those who strictly adhered to the protocol, particularly in more challenging cases in tertiary care settings. The inclusion of participants aged 18 to 50 years could represent an additional limitation. However, given the pilot nature of our study, this age range was chosen considering the peak incidence and prevalence of FM in middle‐aged individuals, as well as the need for participants with relatively greater technologic proficiency.^1^, ^3^ Expanding enrollment to older individuals in future trials may improve the generalizability of the findings.

Despite these limitations, the study demonstrates the feasibility and the promising potential of the IVR‐BF approach for FM treatment and suggests that IVR can enhance biofeedback efficacy by fostering a more engaging and relaxing experience. Our results, although preliminary, endorse the FDA formal recognition of the emerging field of immersive therapeutics, encouraging the use of IVR in chronic musculoskeletal pain.^33^

In conclusion, the multisensor IVR‐BF system appears to provide promising preliminary clinical advantages in pain relief and quality of life for patients with FM in tertiary care settings. Given the favorable feasibility data and high patient satisfaction with minimal adverse events, these encouraging results support the need for larger‐scale studies to further evaluate the efficacy of IVR‐BF across diverse populations and to optimize treatment protocols.

## AUTHOR CONTRIBUTIONS

All authors contributed to at least one of the following manuscript preparation roles: conceptualization AND/OR methodology, software, investigation, formal analysis, data curation, visualization, and validation AND drafting or reviewing/editing the final draft. As corresponding author, Prof Quartuccio confirms that all authors have provided the final approval of the version to be published and takes responsibility for the affirmations regarding article submission (eg, not under consideration by another journal), the integrity of the data presented, and the statements regarding compliance with institutional review board/Declaration of Helsinki requirements.

## Supporting information


**Disclosure form**.

**Appendix S1:** Supplementary Information
