# Supplementary material for: Efficacy of Immersive Virtual Reality Combined With Multisensor Biofeedback on Chronic Pain in Fibromyalgia: A Pilot Randomized Controlled Trial
Source: ACR Open Rheumatol. 2025 May 15;7(5):e70048. doi: 10.1002/acr2.70048 (PMC12080186; doi:10.1002/acr2.70048)
Supplement: Supplementary file 2 — Appendix S1: Supplementary Information [file ACR2-7-e70048-s002.pdf]

Supplementary materials

Table of contents

FIGURES

Supplementary Figure S1 …………………………………………………………………… 2

Supplementary Figure S2 …………………………………………………………………… 2

Supplementary Figure S3 …………………………………………………………………… 3

Supplementary Figure S4 …………………………………………………………………… 3

Supplementary Figure S5 …………………………………………………………………… 4

Supplementary Figure S6 …………………………………………………………………… 4

Supplementary Figure S7 …………………………………………………………………… 5

TABLE

Supplementary Table S1 …………………………………………………………………… 6

Supplementary Table S2 …………………………………………………………………… 6

Supplementary Table S3 …………………………………………………………………… 7

Patient's satisfaction questionnaire about the IVR experience……8

STUDY PROTOCOL

FIGURES

Supplementary Figure S1. **Overview of the study design.**

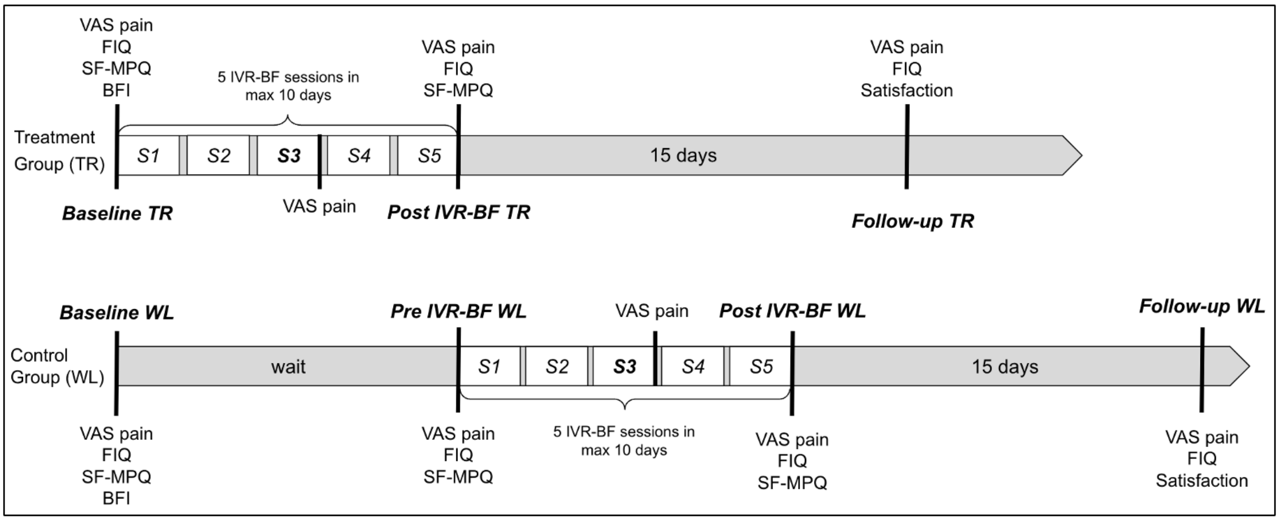

Legend: IVR-BF= Immersive Virtual Reality Biofeedback; VAS= Visual Analogue Scale; FIQ= Fibromyalgia Impact Questionnaire; SF-MPQ= Short-Form McGill Pain Questionnaire; BFI= Big Five Inventory; TR= Treatment; WL= Wait-list.

Supplementary Figure S2. **Design of the between-subjects study.**

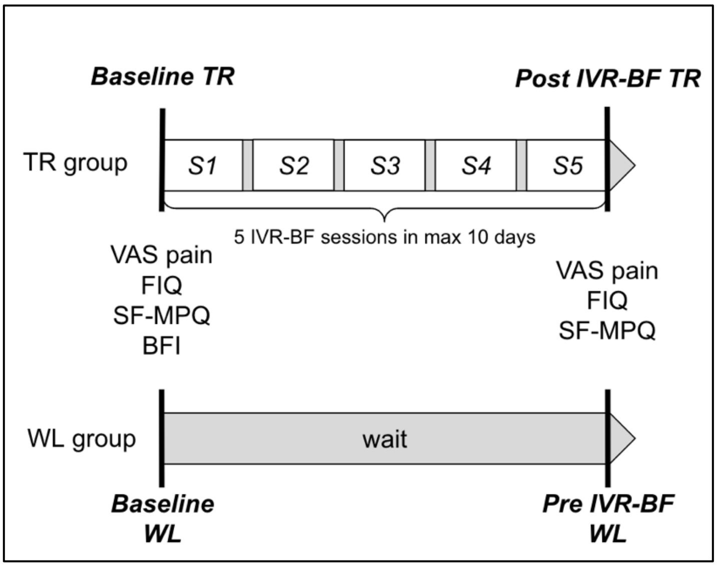

Legend: IVR-BF= Immersive Virtual Reality Biofeedback; VAS= Visual Analogue Scale; FIQ= Fibromyalgia Impact Questionnaire; SF-MPQ= Short-Form McGill Pain Questionnaire; BFI= Big Five Inventory; TR group= Treatment group; WL group= Wait-list group.

Supplementary Figure S3. **Design of the within-subjects study.**

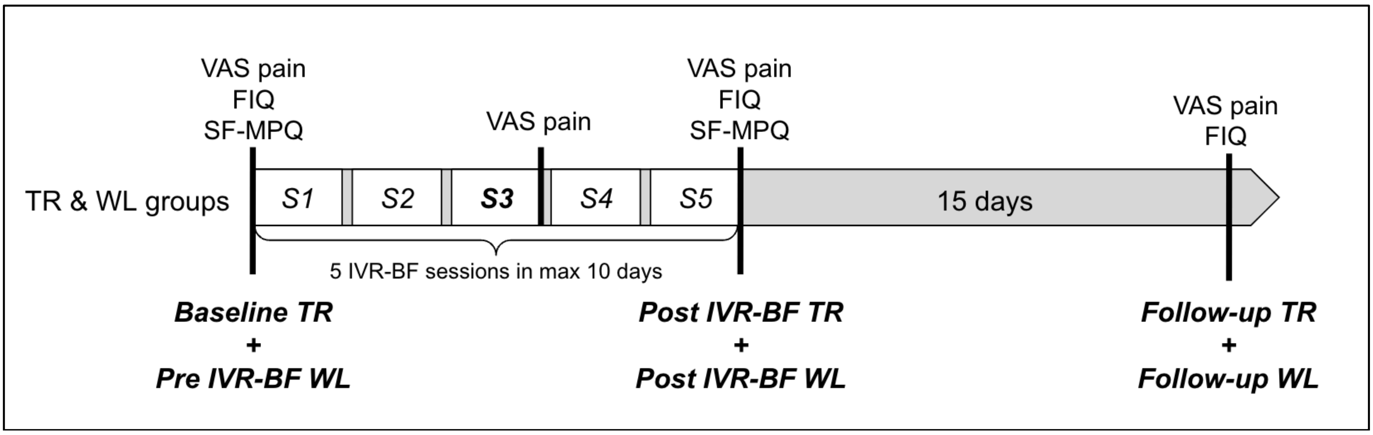

Legend: IVR-BF= Immersive Virtual Reality Biofeedback; VAS= Visual Analogue Scale; FIQ= Fibromyalgia Impact Questionnaire; SF-MPQ= Short-Form McGill Pain Questionnaire; BFI= Big Five Inventory; TR group= Treatment group; WL group= Wait-list group.

Supplementary Figure S4. **Virtual environment of the IVR-BF system as seen from the user's viewpoint.**

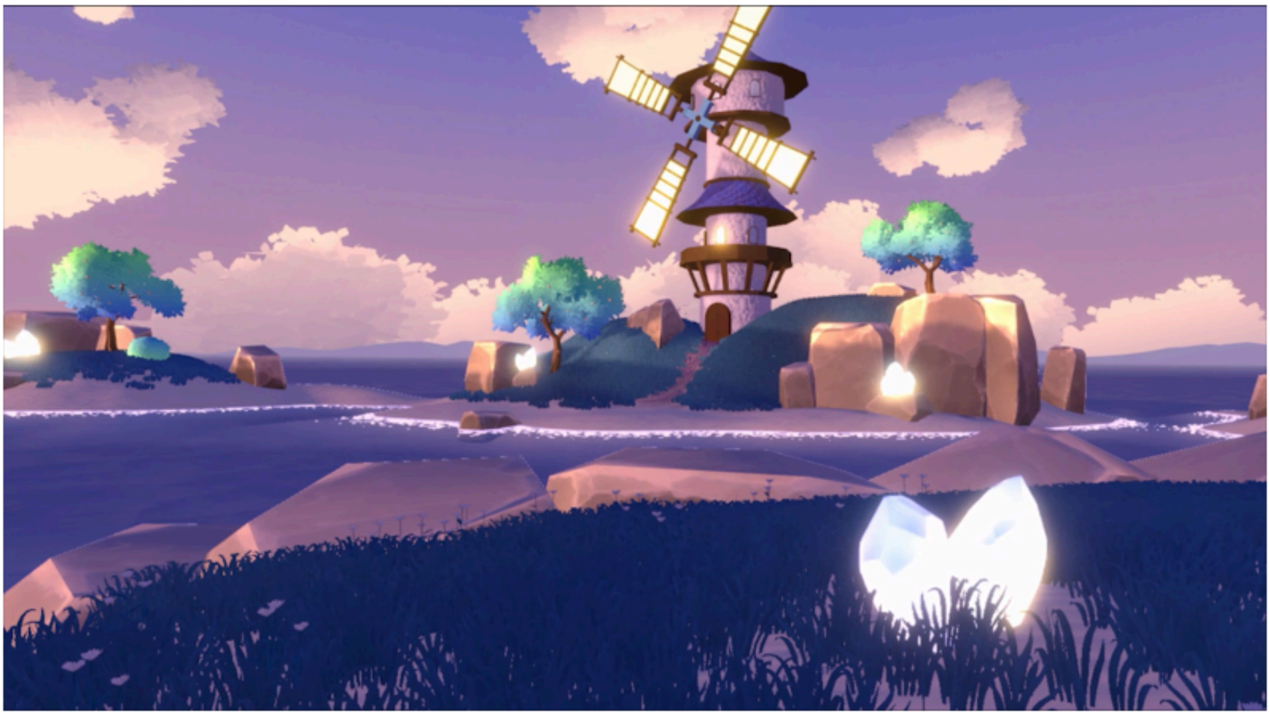

Supplementary Figure S5. **Change in sensory (a), affective (b), total (c) and PPI (d) SF-MPQ score in the treatment group (group TR) compared to the control group (group WL).**

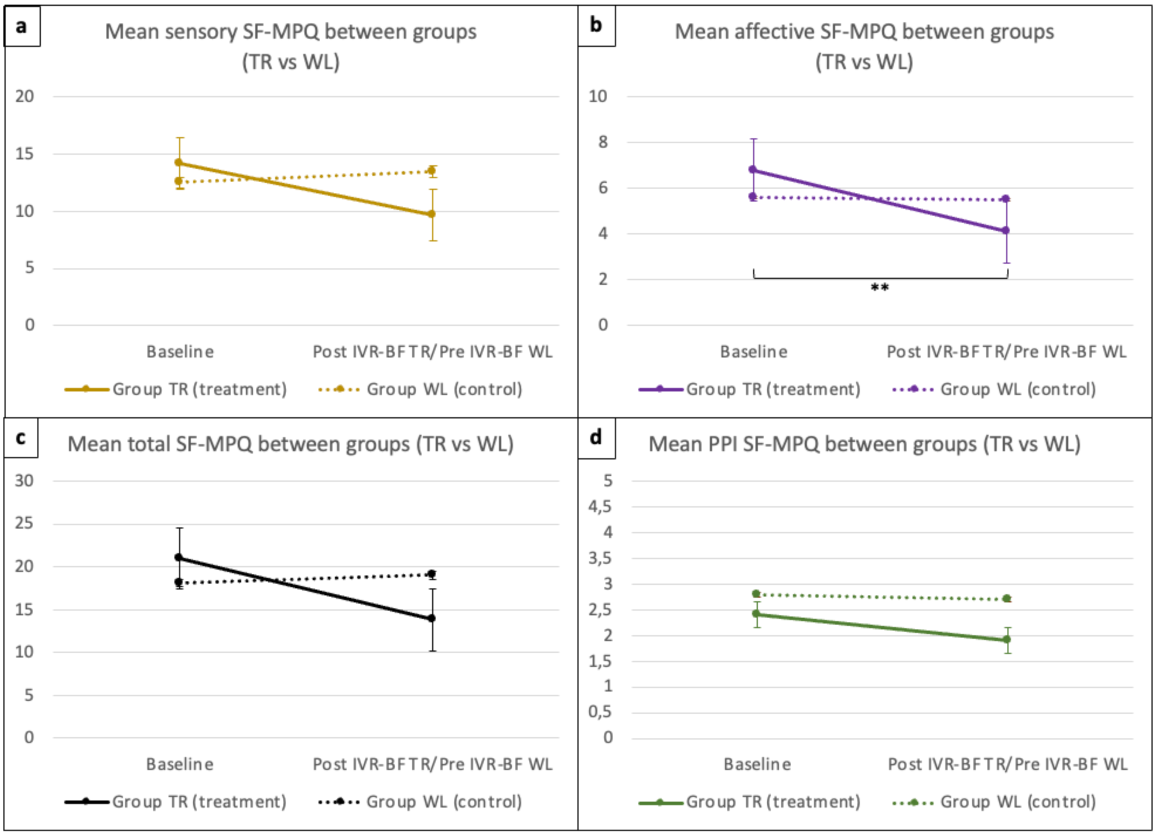

A statistically significant improvement in affective SF-MPQ score was observed in the treatment group (group TR) from baseline to the end of the IVR-BF sessions [**=p<0.01]. Statistical test performed: Repeated measures ANOVA. Legend: IVR-BF= Immersive Virtual Reality Biofeedback; SF-MPQ= Short-Form McGill Pain Questionnaire.

Supplementary Figure S6. **Adverse events (AE) reported during the IVR-BF intervention period (after each session from S1 to S5). No persistent disturbances were reported during follow-up period.**

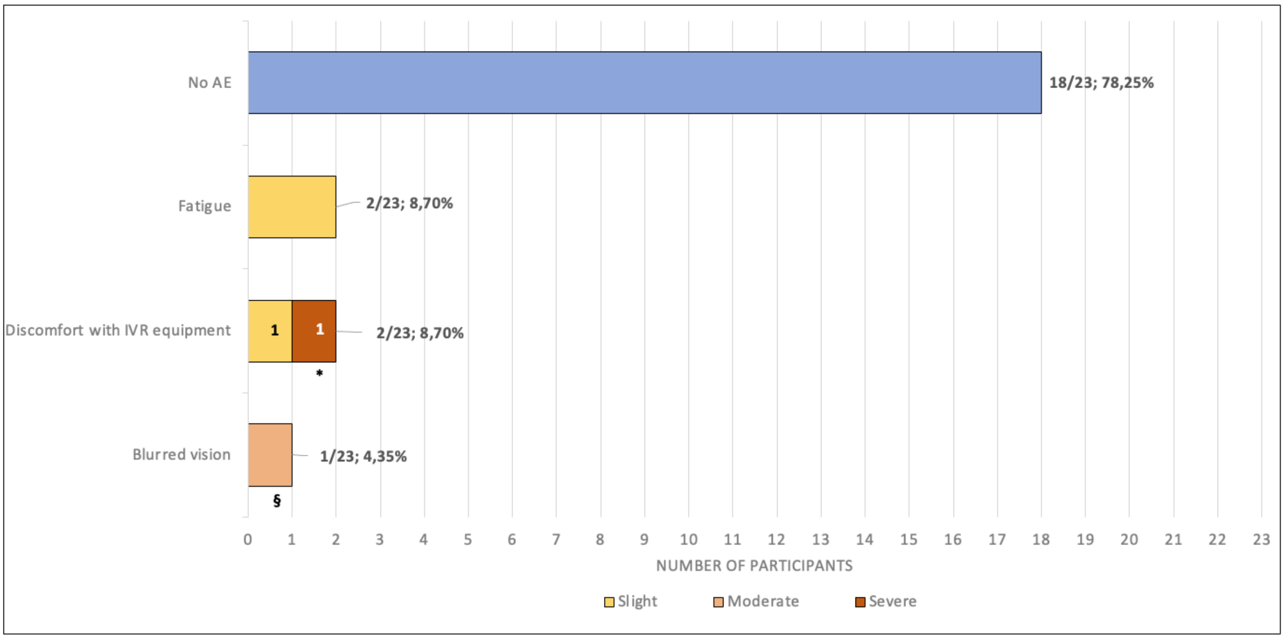

Legend: *= this patient withdrew from the study after S1 due to a strong feeling of constriction associated to the equipment; §= this patient withdrew from the study after S3 due to moderate blurred vision.

Supplementary Figure S7. **Complete report of the responses obtained from the patients' satisfaction questionnaire about the IVR experience, categorized by Question 1 (A), Question 2 (B), Question 3 (C), and Question 4 (D)**

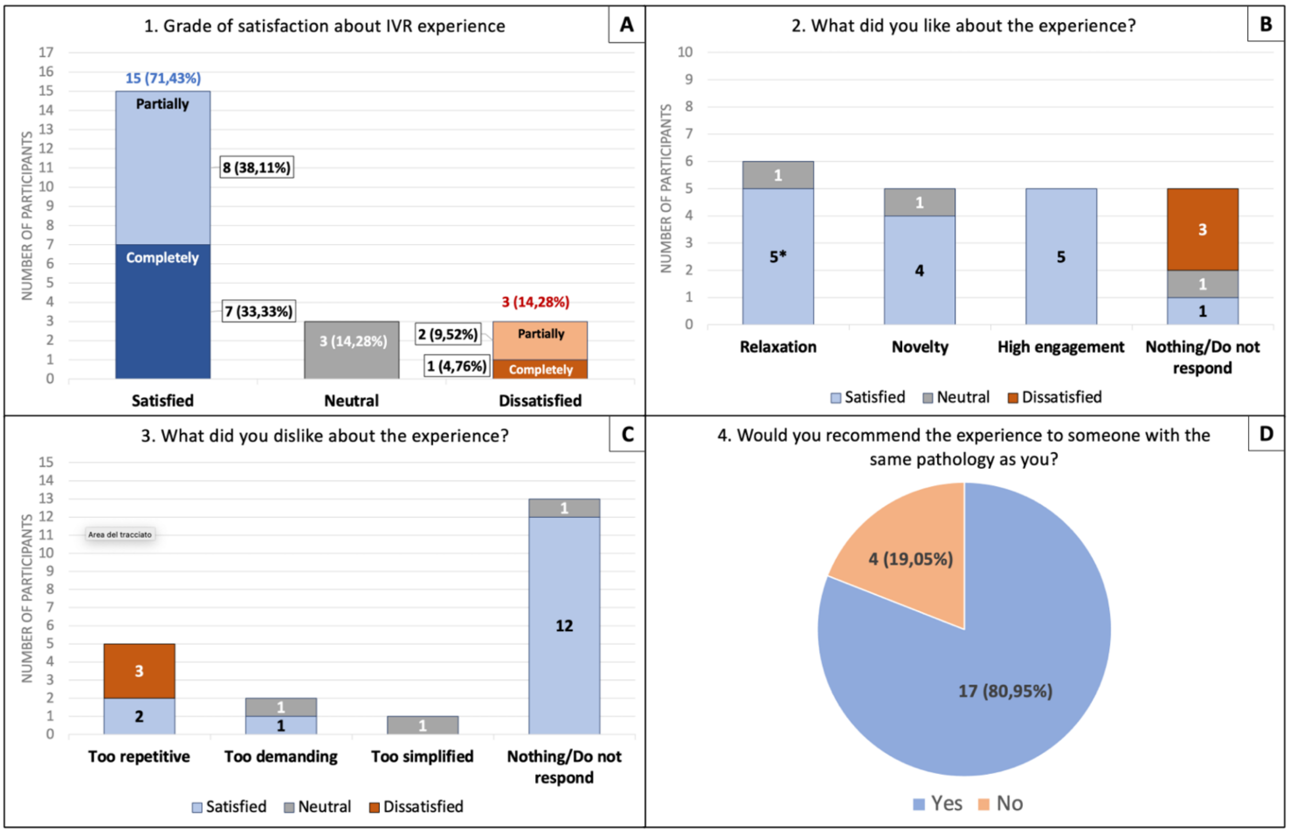

A) **<u>Question 1:</u>** Fifteen patients reported being satisfied with the IVR experience, with 8 indicating partial satisfaction and 7 complete satisfaction. Three patients expressed dissatisfaction, with 2 partially dissatisfied and 1 completely dissatisfied. Three patients remained neutral.

B) **<u>Question 2:</u>** Five satisfied patients highlighted the relaxation and high engagement of the IVR experience, while four satisfied patients emphasized its novelty. One neutral patient appreciated the relaxation, and another valued the novelty provided by the IVR experience. All dissatisfied patients, one neutral patient, and one satisfied patient did not answer the question or found nothing appealing about the IVR experience.

C) **<u>Question 3:</u>** Nearly all patients satisfied with the IVR experience (12/15) found nothing they disliked, apart from two who described it as too repetitive and one as too demanding. One neutral patient found nothing she disliked, while another considered IVR experience too simple, and one found it too demanding. All dissatisfied patients described the experience as overly repetitive.

D) **<u>Question 4:</u>** Beyond the reported satisfaction, 17 patients would recommend the treatment to another person with FM, while only 4 would not.

Legend: *= one patient reported disappearance of musculoskeletal pain during the IVR-BF intervention period.

TABLES

Supplementary Table S1. Variables considered for minimization stratification

Age	//
Sex	Female<br>Male
Comorbidity	None<br>Spondylarthrosis/peripheral arthrosis<br>Depression<br>Migraine<br>Chronic pelvic pain
Marital status	Single<br>Married<br>Divorced<br>Widow
Employment	Unemployed<br>Physically demanding job<br>Sedentary job
Pharmacologic therapy	None<br>Acetaminophen/NSAIDs<br>Opioids/Cannabinoids<br>Antidepressants (duloxetine, amytriptiline)/antiepileptic<br>Muscle relaxants
Non-pharmacologic therapy	None<br>Physical activity<br>Balneotherapy<br>Ozone therapy<br>Cognitive behavioral therapy<br>Meditation<br>Acupuncture

Supplementary Table S2. Results of 2x2 mixed design ANOVA for the between-subjects study and results of repeated measures ANOVA for the within-subjects study

**Study**	**Measure**	**Main effect**			**Interaction**		
		**F**	**p**	**η_p^2**	**F**	**p**	**η_p^2**
Between-subjects	VAS pain	F(1,18)=12.15	0.003	0.40	F(1,18)=20.31	<0.001	0.53
	FIQ	F(1,18)=34.91	<0.001	0.66	F(1,18)=40.77	<0.001	0.69
	SF-MPQ sensory	F(1,18)=1.21	0.285	0.06	F(1,18)=3.00	0.101	0.14
	SF-MPQ affective	F(1,18)=5,65	0.029	0.24	F(1,18)=4,87	0.041	0.24
	SF-MPQ total	F(1,18)=2.32	0.145	0.11	F(1,18)=3.83	0.066	0.18
	SF-MPQ PPI	F(1,18)=2.84	0.109	0.14	F(1,18)=1.26	0.28	0.07
Within-subjects	VAS pain	F(1.98, 37.55)=10.34	<0.001	0.35			
	FIQ	F(2,38)=19.67	<0.001	0.51			
	SF-MPQ sensory	F(1,19)=13.52	0.002	0.42			
	SF-MPQ affective	F(1,19)=17.34	0.001	0.48			
	SF-MPQ total	F(1,19)=16.26	0.001	0.46			
	SF-MPQ PPI	F(1, 19)=5.94	0.025	0.24			

Legend: VAS= Visual Analogue Scale; FIQ= Fibromyalgia Impact Questionnaire; SF-MPQ= Short-Form McGill Questionnaire; PPI= Present Pain Index

Supplementary Table S3. **Pearson's correlation results between BFI and VAS pain score, FIQ score, SF-MPQ affective, sensory, total scores and PPI.**

**Measure**	**Extraversion**			**Agreeableness**			**Conscientiousness**			**Neuroticism**			**Openness**		
	**r**	**n**	**p**	**r**	**n**	**p**	**r**	**n**	**p**	**r**	**n**	**p**	**r**	**n**	**p**
Δ VAS pain	0.24	20	0.308	019	20	0.685	0.29	20	0.220	-0.10	20	0.680	-0.05	20	0.835
Δ FIQ	-0.03	20	0.903	0.01	20	0.977	0.18	20	0.45	0.34	20	0.142	-0.33	20	0.162
Δ SF-MPQ sensory	-0.10	20	0.674	0.03	20	0.891	0.24	20	0.302	0.01	20	0.966	-0.03	20	0.918
Δ SF-MPQ affective	-0.23	20	0.340	-0.05	20	0.832	0.09	20	0.703	-0.01	20	0.979	-38	20	0.096
Δ SF-MPQ total	-0.15	20	0.538	0.01	20	0.975	0.21	20	0.382	0.01	20	0.982	-0.14	20	0.550
Δ SF-MPQ PPI	-0.06	20	0.811	-0.07	20	0.755	0.06	20	0.791	0.08	20	0.754	-0.29	20	0.210

Legend: VAS= Visual Analogue Scale; FIQ= Fibromyalgia Impact Questionnaire; SF-MPQ= Short-Form McGill Questionnaire; PPI= Present Pain Index

PATIENTS' SATISFACTION QUESTIONNAIRE ABOUT THE IVR EXPERIENCE

In the following questionnaire we would like to evaluate your overall satisfaction with the IVR experience. We will ask you to briefly answer some closed and open questions. The questionnaire will be completely anonymous.

1. What is your grade of satisfaction about the experience?
   (1= completely negative, 2= partially negative, 3= neutral, 4= partially positive, 5= completely positive)
   - o 1
   - o 2
   - o 3
   - o 4
   - o 5

2. What did you like about the experience?
   (Free text)

   ____
   ____
   ____
   ____
   ____

3. What did you dislike about the experience?
   (Free text)

   ____
   ____
   ____
   ____
   ____

4. Would you recommend the experience to someone with the same pathology as you?
   - o Yes
   - o No

N.B. This questionnaire was translated from Italian into English to reader's convenience

STUDY PROTOCOL

STUDY TITLE

Effectiveness of virtual reality (VR) in non-oncologic chronic musculoskeletal pain: a randomized controlled trial in Fibromyalgia

PROMOTOR

Rheumatology Division, Department of Medicine, University of Udine – ASUFC.

Principal Investigator: Prof. Luca Quartuccio, MD, PhD

Sub-investigators: Prof. Luca Chittaro, PhD; Marta Serafini, PhD student; Simone Longhino, MD; Sofia Cacioppo, MD.

PARTICIPATING CENTERS

- Human-Computer Interaction Laboratory – Department of Mathematics, Computer Science and Physics (DMIF) - University of Udine (Prof. Luca Chittaro, PhD; Marta Serafini, PhD student)
- Rheumatology Division, Department of Medicine, University of Udine – ASUFC (Prof. Luca Quartuccio MD, PhD; Simone Longhino, MD; Sofia Cacioppo, MD)

INTRODUCTION

Virtual Reality (VR) is defined as a digital technology in which sensory experiences (e.g., visual, auditory, tactile, and olfactory stimuli) are artificially created, prompting users to manipulate objects within the virtual environment (1). Depending on the level of user immersion in this digital reality, VR can be classified as immersive or non-immersive. Immersive VR is capable of completely isolating the participant from the real world, offering a greater sense of presence for the user, both perceptually and psychologically, while in non-immersive VR, subjects are influenced by the surrounding environment (2). In the medical field, VR, used as a method for physical and cognitive training, has shown benefits in various contexts (3). Thanks to its ability to simulate real-world situations and cognitive and motor tasks in a safe environment, completing VR activities represents a rewarding form of therapy for patients needing motor rehabilitation or musculoskeletal pain management. The use of VR in the medical field is indeed very broad, ranging from the neurological field (stroke, spinal cord injury, neuropathic pain, Parkinson's, multiple sclerosis) (4), pediatric field (cerebral palsy) (5), and psychiatric field (phobias and post-traumatic stress disorder). Recently, VR has also been proposed as an innovative method in the non-pharmacological treatment of chronic pain, incorporating activities such as physical exercise and biofeedback (BF) into an immersive and potentially enjoyable environment for the patient. This is particularly true for fibromyalgia, a rheumatic disorder characterized by chronic musculoskeletal pain. Indeed, few studies have shown that the use of VR systems in the rehabilitation of patients with FM can reduce pain, fatigue, and static balance problems, improving exercise capacity, mobility, balance, and overall health-related quality of life (HRQoL), as well as the ability to perform daily living activities with a high degree of tolerability (6,7). However, currently, the literature has almost exclusively explored non-immersive VR systems without BF in the context of FM. The use of immersive systems could increase patient engagement, and the introduction of BF could have a synergistic effect, enhancing positive outcomes for patients. A promising indication in this regard is provided by the pilot study by Venuturupalli et al. in which patients with various inflammatory rheumatic disease (excluding patients with fibromyalgia) reported a statistically significant

CERTIFICAZIONE DI SISTEMA QUALITÀ DNV·GL ISO 9001

reduction in pain after immersive VR-BF (IVR-BF), with high satisfaction regarding the experience, spontaneously stating that they had achieved a greater degree of relaxation and calm (8).

STUDY RATIONALE

Although numerous pharmacological and non-pharmacological treatments for non-cancer musculoskeletal chronic pain are available today, its therapeutic management still presents many challenges. A multimodal and multidisciplinary approach plays a key role, leveraging the integration of pharmacological and non-pharmacological interventions such as psycho-cognitive-behavioral, educational, and rehabilitative techniques. However, in some cases, this approach proves insufficient in reducing the impact on the affected individual, particularly in terms of pain. This leads to the risk of painkiller abuse, especially opioids, perpetuating a vicious cycle characterized by the consumption of an increasing number of drugs at higher doses without significant benefits. It thus becomes evident that new therapeutic strategies, particularly non-pharmacological ones like IVR-BF, may be useful in implementing the holistic approach that is essential for the proper management of non-cancer musculoskeletal chronic pain, in particular for the pain related to fibromyalgia.

STUDY OBJECTIVES

a. **Primary endpoint**

To test the hypothesis of a significant reduction in pain intensity, as evaluated by the VAS pain score, of at least 30% in participants with non-cancer musculoskeletal chronic pain, subjected to a combined intervention of IVR-BF compared to participants not subjected to this treatment.

b. Rationale

Non-cancer musculoskeletal chronic pain, particularly fibromyalgia pain, has a significant impact both from an individual perspective, due to the low quality of life of the affected subjects, and from a social perspective, due to the direct and indirect costs it entails (9,10). Pain is strongly influenced by stress, anxiety, and depression (11-13). For this reason, the rationale behind the use of IVR is to enhance those biofeedback techniques that have already shown a promising role in reducing anxiety and pain, although they are not currently indicated for the management of fibromyalgia pain (14,15). The 30% threshold value was chosen as it is one of the main objectives for pain reduction according to the IMMPACT guidelines (16).

c. Secondary endpoints

Secondary outcomes comprise: a) mitigation of FM impact on daily life, defined as a significant decrease in the FIQ score; b) improvement of sensory and emotional aspects of pain, assessed as reductions in sensory, affective, and total SF-MPQ scores. Moreover, a longitudinal analysis will be conducted to examine the temporal trends in the VAS pain scale, the SF-MPQ, and the FIQ score. This analysis will encompass all participants in the study, starting from baseline for the TR group, and from pre IVR-BF for the WL group, and reaching follow-up assessment in both groups. Potential correlations between specific BFI personality traits and the response to IVR-BF treatment will be also investigated.
Furthermore, we will perform an analysis of the physiological biofeedback data collected by the IVR-BF system during the performance of Task1 (see *Description of virtual reality experience* for further details) in each session. We consider only Task1 since this task is the only task requiring

CERTIFICAZIONE DI SISTEMA QUALITÀ DNV·GL ISO 9001

the participants to perform a specific physiological activity (slow and deep breathing), with the VE changing to reinforce that breathing activity. Finally, we will evaluate the tolerability of the treatment, recording any adverse events occurring during IVR-BF treatment.

PARTICIPANT SELECTION

a. Inclusion criteria

Volunteers aged between 18-50 years who suffer from non-cancer musculoskeletal chronic pain and meet the 2016 Modified ACR Diagnostic Criteria for Fibromyalgia will be included in the analysis.

b. Exclusion criteria

- Concomitant diagnosis of major psychiatric disorders except for anxiety-depressive disorder;
- Concomitant diagnosis of neurological disorders;
- Concomitant diagnosis of inflammatory rheumatologic diseases that may cause chronic pain;
- Concomitant diagnosis of severe heart diseases;
- Concomitant diagnosis of epilepsy;
- Concomitant diagnosis of issues related to reality perception;
- Substance addiction.

c. Rationale for exclusion criteria

These criteria refer to participants who are unlikely to benefit from the VR experience due to their underlying conditions.

STUDY DESIGN

a. Protocol description and randomization

This is a single-center open-label randomized controlled trial conducted in a tertiary care referral center for non-oncologic chronic fibromyalgia pain.

The phases of the study will be as follows:

- **Participant selection:** there are no time limits for the selection of participants, who will be recruited from the Rheumatology Clinic of Udine (ASUFC). Participants will undergo randomization through a computerized system. There will be no time limits for randomization.
- **Randomization**: Participants will be randomly assigned in a 1:1 ratio to two study groups: a treatment group (TR), which will receive the IVR-BF intervention immediately after randomization, and a control group (WL), which will receive the same intervention only after the TR group completed it. Each group will be evaluated through questionnaires up to 15 days after the end of the last IVR-BF session. The randomization will be performed using an automated software (StudyRandomizer.com) employing a minimization algorithm to balance the following variables: age, gender, marital status, comorbidities, employment, and concomitant pharmacological and non-pharmacological treatments. The randomly assigned allocation of each participant will be masked and revealed to the investigators just before the baseline assessment. The participants will be blinded to the group allocation, while the investigators will not, as directly involved in administering the IVR-BF intervention.
- **Treatment**: IVR-BF will be administered to volunteers once a day for 5 days (preferably but not necessarily consecutive) within a maximum period of 10 days, at the Rheumatology Clinic, Santa Maria della Misericordia Hospital, Udine. The TR group will undergo IVR-BF

CERTIFICAZIONE DI SISTEMA QUALITÀ DNV·GL ISO 9001

immediately after randomization, while the WL group will undergo IVR-BF only after the treatment group has completed their sessions.

- **Follow up**: follow-up will begin at randomization and will continue until the end of the study according to the procedures outlined below.

b. Study procedure

Each participant in the TR group will undergo five sessions of IVR-BF intervention (maximum one session per day), preferably but not necessarily consecutively within a maximum timeframe of ten days. After the completion of all IVR-BF sessions for each participant in the TR group, participants in the WL group will receive the IVR-BF intervention following the same procedure. Each participant in both groups will then be monitored over time for up to 15 days after the end of the last IVR-BF session. For each IVR-BF session, the participant will be comfortably seated in an armchair and equipped with a Meta Quest 2 headset (Meta, USA). A Quest Touch controller (Meta, USA) for use with the right hand will be provided for a brief interaction with the virtual environment (VE) that will be required just once during the experience. To capture the physiological data for the biofeedback, the participant will wear a photoplethysmograph on the distal phalanx of the middle finger of the left hand to measure heart rate, two electrodes on the center of the palm and the carpus of the left hand to measure skin conductance, and an elastic girth sensor around the abdomen to assess respiratory activity. All physiological sensors (Thought Technology, Canada) will be recorded in real-time using a Thought Technology ProComp Infiniti encoder (Thought Technology, Canada) at a sampling rate of 10 Hz. Each IVR-BF session will last 15 minutes and will start with an initial phase that immerses the participant in a neutral VE representing a living room, while the baseline of physiological activity will be recorded for three minutes. The participant will then perform diaphragmatic breathing for one minute, guided by a voice-over that will provide instructions. Next, the participant will experience a natural VE as described in *Description of virtual reality* below. The participant will be required to perform two tasks that change the appearance of the VE: 1) clearing the fog in the VE through slow, deep breathing (Task1); 2) making the night fall by deeply relaxing, thereby decreasing skin conductance (Task2). The two tasks will each last three minutes. If the participant fails to complete them within this time frame, the system will automatically complete them. During all the experience, the participant will control different graphical elements of the environment through biofeedback as described more specifically below. During the study, questionnaires will be administered at several time points, as illustrated by the detailed timeline in Figure A. The questionnaires will include: 1) Visual Analogue Scale for pain, from 0 to 100 (VAS pain), to assess pain intensity; 2) Fibromyalgia Impact Questionnaire (FIQ), to assess the impact of FM on daily life; 3) Short-form McGill Pain Questionnaire (SF- MPQ), to assess sensory and affective aspects of pain; 4) Big Five Inventory (BFI), to assess personality traits. At the end of the study, satisfaction questionnaires about the IVR experience will be administered to all participants.

The physiological data collected during IVR-BF sessions will include: 1) skin conductance (SC); 2) respiratory rate (RR); 3) heart rate (HR); 4) heart rate variability (HRV), expressed as Root Mean Square of Successive Differences (RMSSD), number of pairs of successive heartbeat intervals differing by more than 50 milliseconds (NN50), and proportion of NN50 divided by the total number of heartbeat intervals (pNN50). These parameters are chosen because they are recognized as physiological indicators of stress and pain. HRV will be recorded at 500 Hz using a blood volume pulse finger clip sensor placed on the distal phalanx of the index finger of the left hand and connected to a BioSignalsPlux encoder (PLUX Biosignals, Portugal). A sampling rate of 500 Hz is recommended for HRV analysis, as lower sampling rates can cause inaccuracies in HRV analysis.

CERTIFICAZIONE DI SISTEMA QUALITÀ DNV·GL ISO 9001

The VR headsets, respiratory monitors, skin conductance sensors, and heart rate monitors used in the study are property of the Human-Computer Interaction Laboratory, Department of Mathematical, Computer, and Physical Sciences (DMIF) at the University of Udine; therefore, the study does not incur additional costs.

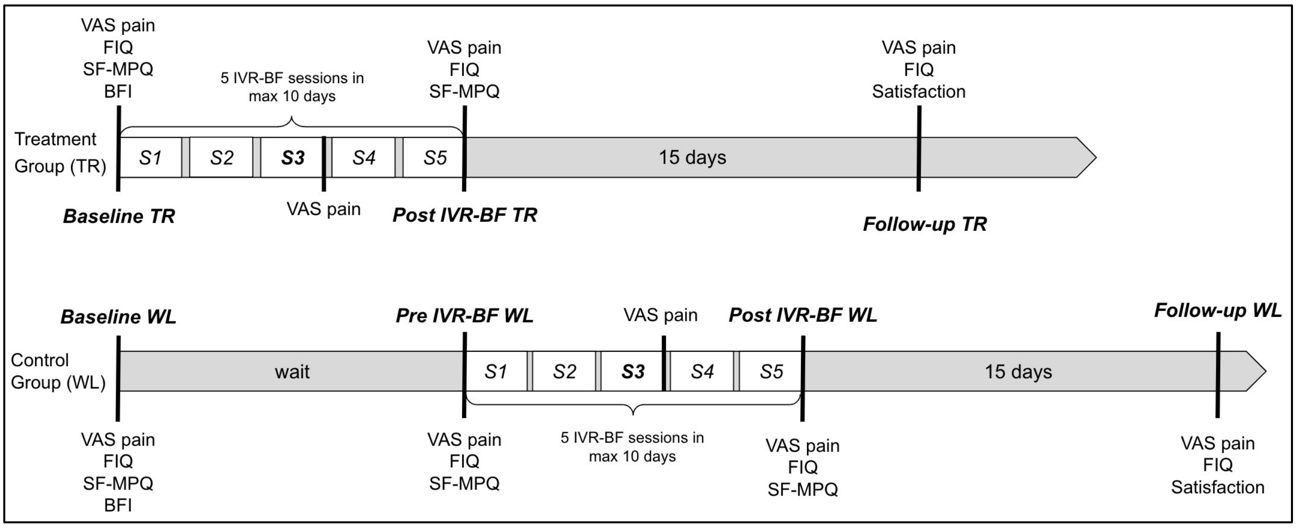

Figure A. Study design with timeline of questionnaire assessment

DESCRIPTION OF VIRTUAL REALITY EXPERIENCE

a. Preparation of the participant for the Virtual Reality experience

The volunteer is comfortably seated in a chair and asked to wear an Oculus Quest 2 headset, which isolates them visually and acoustically from the surrounding environment. At the same time, a photoplethysmograph is placed on the distal phalanx of the left hand and connected to a device (BioSignalPlux) capable of measuring the participant's heart rate. On the palm and dorsal side of the right wrist, two electrodes are placed to measure skin conductance. An elastic sensor is wrapped around the waist to assess respiratory activity.

b. Description of the virtual reality

Before the actual virtual experience begins, the participant will be immersed in a neutral environment (a virtual living room) for three minutes, during which the system will record baseline physiological values (heart rate, skin conductance, and standard chest excursion). Subsequently, a guided breathing training will be provided, where a voice-over will explain how to perform slow, deep diaphragmatic breathing to evaluate maximum chest excursion during exhalation and inhalation. Visually, the user will see their breath mapped with biofeedback on a lotus flower placed on a table in the living room. Following this, the participant will be immersed in the actual virtual reality experience, which consists of a stylized visual representation of a coastal environment with a long, narrow beach facing the sea. Three islands of various sizes will be near the shore, and the largest island will have a windmill.

The user will be virtually placed on a wooden bench with a bush on the right. The virtual environment (Virtual Environment, VE) will be called 'Archipelago of Crystals' due to the numerous crystals scattered around. The experience will start around sunset, and the VE will initially be covered in fog. Throughout the experience, relaxing background music and the sounds of the wind, sea, and windmill blades will be played. A recorded voice-over will introduce the story and guide the participant through tasks (exploring the surrounding bush, releasing fireflies in the VE, clearing the fog, and making night fall) through various guided exercises (deep breathing, relaxation, etc.). The biofeedback mechanism will ensure that changes in the participant's physiological signals during the experience will influence events within the VE (for example, the rotation of the windmill blades will be connected to breathing so that slow, deep breathing will clear the fog in the VE). At the end of the approximately 15-minute experience, the narrator will announce that the experience has concluded.

c. Structure of the Virtual Experience

The VR experience will be organized into 5 phases:

- **Calibration**: In the first phase, the system will be calibrated to the participant's breathing amplitude, detecting maximum and minimum chest expansion values using the belt sensor placed around the waist. To this end, the voice-over will ask the user to take three deep breaths. For 60 seconds, the system will monitor the user's breathing, recording the maximum and minimum expansion values detected by the sensor.
- **Clearing the Fog** (Task1): The participant will be instructed by the narrator on how to breathe slowly and deeply with the diaphragm and will be encouraged to do so to clear the VE of fog. This activity will last up to 3 minutes, after which the fog will automatically disappear.

CERTIFICAZIONE DI SISTEMA QUALITÀ
DNV·GL
ISO 9001

- **Interacting with the Bush**: The user will hear the rustling of leaves coming from the bush on their right, and the voice-over will invite them to touch it. When the user touches the
- bush, fireflies will emerge and slowly fly near the user. If the user does not interact with the bush within 20 seconds, the fireflies will automatically emerge from the bush to allow the user to proceed to the next phase.
- **Making Night Fall** (Task2): The voice-over will inform the user that sunset is approaching and will ask them to deeply relax to bring on the night. The activity will last for three minutes, during which time the night will automatically take over if the environment is not already in full nighttime conditions**.**
- **Finale**: This phase marks the end of the experience. The background music will change from calm to livelier, the fruits on the trees will light up, particles of light will gently rise from all the crystals, and the fireflies will begin to fly toward the sky, drawing luminous trails in the night. The voice-over will bid farewell to the user and invite them to return soon to the Archipelago of Crystals.

d. Biofeedback systems

The biofeedback system implemented in the IVR will include:

- **Breathing**: To determine if the user is breathing slowly and deeply, breathing data will be normalized during the first phase of the experience so that maximum and minimum chest expansion correspond to 1 and 0, respectively. If chest expansion exceeds a threshold of 0.7, inhalation is considered deep; conversely, if it is below a threshold of 0.45, exhalation is considered deep. The user will control the wind in the VE through their breathing activity throughout the experience. Each time the user inhales, the leaves of the trees and bushes will slow down their oscillation; conversely, when the user exhales, they will oscillate faster up to a maximum threshold. If the user holds their breath, the oscillation speed will decrease until the leaves stop moving completely. Each exhalation will also be accompanied by the sound of the wind blowing, with the volume increasing if the exhalation is deep. The windmill blades will behave similarly, taking into account the respiratory rate: the rotation speed of the blades will slow down during inhalations (the longer the inhalation, the slower the speed) and increase during exhalations (the faster the exhalation, the higher the speed). Additionally, since the windmill blades accelerate during exhalation, the volume and pitch of the gear sounds will also increase. To indicate whether the user is performing slow, deep breathing, the windmill blades will light up pink during deep inhalation and yellow during deep exhalation. If the user breathes too quickly, the windmill blades will jam: they will slow down abruptly, oscillate, and emit red sparks. During the second phase of the virtual experience, if the user takes a deep inhalation, the subsequent exhalation will reduce the fog density and increase the distance of the fog from the user. This change is negligible and insufficient to clear the environment of fog if the user's exhalation is not deep. Therefore, the environment can only be cleared by maintaining slow, deep breathing over time. If the user's breathing is not deep or slow, the amount and density of the fog will increase.
- **Skin Conductance**: The rate of change in conductance values will be used in the fourth phase of the virtual experience to modify ambient lighting: if the rate of change in conductance is negative, indicating greater relaxation of the participant, the position of the sun will lower toward the horizon, and the moonlight will become increasingly bright. Conversely, if the rate of change in conductance is positive, the sun's position will rise toward the zenith, increasing in intensity and decreasing the moonlight.

CERTIFICAZIONE DI SISTEMA QUALITÀ DNV·GL ISO 9001

- **Heart Rate**: The participant's heart rate will affect the frequency with which the fireflies blink: a lower heart rate will result in a slower blinking frequency, while a higher heart rate will make the fireflies blink more frequently.

e. **Study duration**
The study is expected to last 30 days.

PARTICIPANTS' SAFETY

According to various studies evaluating VR, this pain management strategy is considered safe and widely accepted (8, 17, 18). In a single study, some participants expressed negative opinions about the weight of the headset, but none reported issues related to motion sickness (8). In our study, one of the lightest headsets on the market (500 grams) will be used. Participants with neurological conditions that may result in excessive sensitivity to light stimuli will be excluded. Additionally, a physician will always be present to supervise the participant during the VR experience, along with a PhD student from the Department of Mathematical, Computer, and Physical Sciences (DMIF) at the University of Udine. It is known that exposure to flashing lights is contraindicated for individuals who may suffer from seizures. A minority of people experience seizures or momentary loss of consciousness when exposed to flashing lights. If any discomfort (such as eye strain, fatigue, nausea) is experienced, the visual sequences should be stopped until the discomfort subsides. For safety reasons, all equipment will not be connected to the hospital's electrical network. If the hospital electrical network must be used, appropriate electrical outlets compatible with the equipment (standard 220V AC outlets) will be ensured.

STATISTICAL PLAN

a. Sample size

To detect a significant effect in the primary endpoint, a sample size of at least 20 participants will be required (10 per group). The sample size will be determined using PASS software (version 19.0.4), with an alpha error of 0.05, a power of 0.8, an expected VAS pain mean score of 67.11 (SD 14.88) at baseline, and an anticipated reduction of the VAS pain score by 30% in the TR group compared to the WL group. The expected mean and standard deviation, and the anticipated reduction of VAS pain scores derive from values reported in studies from two reviews demonstrating the efficacy of duloxetine, pregabalin, and milnacipran in FM management (19, 20).

b. Statistical analysis

Firstly, a between-subjects analysis to compare the results from the two groups will be performed. VAS, SF-MPQ, and FIQ scores will be submitted to a 2x2 mixed design ANOVA, after checking that its assumptions are met. Group (TR and WL) will serve as the between-subject variable, and time of measurement (before and after the treatment period of the TR group) will serve as the within-subject variable. As suggested by Cohen, if the ANOVA reveals a significant main effect, each simple effect will be analyzed using Bonferroni correction, considering the effects of the group separately at each time of measurement. Then, for the scores where significant differences are observed between the groups, the percentage change from baseline to post-treatment will be calculated for both groups and compared with a Student's t-test, after checking its assumptions.

CERTIFICAZIONE DI SISTEMA QUALITÀ DNV·GL ISO 9001

Next, data from the two groups will be aggregated and treated as a single group. A repeated-measures ANOVA will be used to compare the questionnaire scores over time, starting from baseline for the TR group and pre-IVR-BF for the WL group, until follow-up assessment for both groups. If Mauchly's test indicates a violated assumption of sphericity, degrees of freedom will be corrected using Greenhouse-Geisser estimates. Moreover, Pearson correlation will be computed to assess possible relationships between the five BFI personality traits and the differences between post-treatment and pre-treatment scores of the VAS pain, SF-MPQ, and FIQ. To preprocess physiological data collected during baseline and Task 1, the NeuroKit Python library will be used to extract the tonic (skin conductance level, SCL) component of skin conductance, and derive the HRV measures (RMSSD, NN50, and pNN50). For each session, the Shapiro-Wilk normality test will be performed on the average of physiological data over the baseline and Task 1 intervals, respectively. If the data are not normally distributed, they will be transformed using a logarithm (base 10) or square root transformation. Extreme outliers will be excluded from the analyses. A repeated-measures ANOVA will then be conducted for each session to compare the baseline and Task 1 averages. All analyses will be conducted using SPSS version 29.0.0.0

DATA MANAGEMENT

a. Confidentiality and assurance standard

All information collected during the study will be managed in an anonymized form. The processing of data necessary to produce the analyses outlined in this protocol will comply with the current privacy regulations (European Privacy Regulation 2016/679 for General Data Protection (GDPR)). All parties involved in this study will maintain strict confidentiality. The handling of personal and sensitive data of volunteers participating in the study will be in accordance with the prevailing regulations (as mentioned above). Only anonymized data of volunteers who have given informed consent for their use for clinical research, epidemiological studies, training, and disease study purposes will be collected via the GE.CO system. Data will be processed in accordance with Authorization No. 9/2016, general authorization for the processing of personal data carried out for scientific research purposes – December 15, 2016, point 2 "Purpose of processing in the medical, biomedical, and epidemiological fields.

b. Publications

The results of the analysis will include only aggregated data that cannot be attributed in any way, either directly or indirectly, to the individual. The sponsor will be responsible for the publication of data derived from the study, as well as the potential dissemination of results through communications at conferences, seminars, and thesis.

CERTIFICAZIONE DI SISTEMA QUALITÀ DNV·GL ISO 9001

BIBLIOGRAPHY

1. Qian, J., McDonough, D. J. & Gao, Z. The Effectiveness of Virtual Reality Exercise on Individual's Physiological, Psychological and Rehabilitative Outcomes: A Systematic Review. *Int. J. Environ. Res. Public. Health* **17**, 4133 (2020).

2. Gulsen, C. *et al.* Effect of fully immersive virtual reality treatment combined with exercise in fibromyalgia patients: a randomized controlled trial. *Assist. Technol. Off. J. RESNA* **34**, 256–263 (2022).

3. Costa, M. T. S. *et al.* Virtual Reality-Based Exercise with Exergames as Medicine in Different Contexts: A Short Review. *Clin. Pract. Epidemiol. Ment. Health CP EMH* **15**, 15–20 (2019).

4. Cassani, R., Novak, G. S., Falk, T. H. & Oliveira, A. A. Virtual reality and non-invasive brain stimulation for rehabilitation applications: a systematic review. *J. Neuroengineering Rehabil.* **17**, 147 (2020).

5. Treadmill Training with Virtual Reality Improves Gait, Balance, and Muscle Strength in Children with Cerebral Palsy. https://www.jstage.jst.go.jp/article/tjem/238/3/238_213/_article.

6. Rs, V. *et al.* Virtual Reality-Based Biofeedback and Guided Meditation in Rheumatology: A Pilot Study. *ACR Open Rheumatol.* **1**, (2019).

7. Polat, M., Kahveci, A., Muci, B., Günendi, Z. & Kaymak Karataş, G. The Effect of Virtual Reality Exercises on Pain, Functionality, Cardiopulmonary Capacity, and Quality of Life in Fibromyalgia Syndrome: A Randomized Controlled Study. *Games Health J.* **10**, 165–173 (2021).

8. Venuturupalli, R. S. *et al.* Virtual Reality-Based Biofeedback and Guided Meditation in Rheumatology: A Pilot Study. *ACR Open Rheumatol.* **1**, 667–675 (2019).

9. Salaffi, F. et al. Development and validation of the self-administered Fibromyalgia Assessment Status: a disease-specific composite measure for evaluating treatment effect. Arthritis Res. Ther. 11, R125 (2009).

10. Bernardy, K., Klose, P., Busch, A. J., Choy, E. H. S. & Häuser, W. Cognitive behavioural therapies for fibromyalgia. Cochrane Database Syst. Rev. CD009796 (2013) doi:10.1002/14651858.CD009796.pub2.

11. Moore, A. R. et al. Fibromyalgia: Moderate and substantial pain intensity reduction predicts improvement in other outcomes and substantial quality of life gain. Pain 149, 360–364 (2010).

12. Michaelides, A. & Zis, P. Depression, anxiety and acute pain: links and management challenges. Postgrad. Med. 131, 438–444 (2019).

13. Świtała, W. W., Szymańska-Adamcewicz, O., Jurga, S., Pilchowska-Ujma, E. & Krakowiak, J. Genetic aspects of pain and its variability in the human population. Ann. Agric. Environ. Med. AAEM 28, 569–574 (2021).

14. Macfarlane, G. J. et al. EULAR revised recommendations for the management of fibromyalgia. Ann. Rheum. Dis. 76, 318–328 (2017).

15. Kothgassner, O. D. et al. Virtual reality biofeedback interventions for treating anxiety : A systematic review, meta-analysis and future perspective. Wien. Klin. Wochenschr. 134, 49–59 (2022).

CERTIFICAZIONE DI SISTEMA QUALITÀ DNV·GL ISO 9001

16. Dworkin RH, Turk DC, Farrar JT, et al. Core outcome measures for chronic pain clinical trials: IMMPACT recommendations. Pain. 2005;113(1-2):9-19. doi:10.1016/j.pain.2004.09.012
17. Tsigarides, J., Shenker, N. G. & MacGregor, A. Ready Patient One: the role of therapeutic virtual reality in the future management of chronic pain. *Rheumatol. Oxf. Engl.* **61**, 482–483 (2022)
18. Herrero, R., García-Palacios, A., Castilla, D., Molinari, G. & Botella, C. Virtual reality for the induction of positive emotions in the treatment of fibromyalgia: a pilot study over acceptability, satisfaction, and the effect of virtual reality on mood. Cyberpsychology Behav. Soc. Netw. 17, 379–384 (2014).
19. Welsch P, Üçeyler N, Klose P, Walitt B, Häuser W. Serotonin and noradrenaline reuptake inhibitors (SNRIs) for fibromyalgia. *Cochrane Database of Systematic Reviews* 2018; **2020**. DOI:10.1002/14651858.CD010292.pub2
20. Arnold LM, Emir B, Pauer L, Resnick M, Clair A. Time to Improvement of Pain and Sleep Quality in Clinical Trials of Pregabalin for the Treatment of Fibromyalgia. *Pain Medicine* 2015; **16**: 176–85.

Udine, 15th June 2023

Principal Investigator and Promoter of the Study
Prof. Luca Quartuccio, MD, PhD

CERTIFICAZIONE DI SISTEMA QUALITÀ
DNV·GL
ISO 9001
